# Assessing the Influence of Urine pH on the Efficacy of Ciprofloxacin and Fosfomycin in Immunocompetent and Immunocompromised Murine Models of *Escherichia coli* and *Klebsiella pneumoniae* Infection in the Lower Urinary Tract

**DOI:** 10.3390/antibiotics13090827

**Published:** 2024-09-01

**Authors:** Soraya Herrera-Espejo, Marta Carretero-Ledesma, Manuel Anselmo Bahamonde-García, Elisa Cordero, Jerónimo Pachón, María Eugenia Pachón-Ibáñez

**Affiliations:** 1 Clinical Unit of Infectious Diseases, Microbiology and Parasitology, Institute of Biomedicine of Seville (IBiS), Virgen del Rocio University Hospital/CSIC/University of Seville, 41013 Seville, Spain; sherrera-ibis@us.es (S.H.-E.); mcarretero-ibis@us.es (M.C.-L.); manselmo.bahamonde.sspa@juntadeandalucia.es (M.A.B.-G.); mpachon-ibis@us.es (M.E.P.-I.); 2 Department of Medicine, School of Medicine, University of Seville, 41004 Seville, Spain; 3 CIBER de Enfermedades Infecciosas, Instituto de Salud Carlos III, 28029 Madrid, Spain; 4 Institute of Biomedicine of Seville (IBiS), Virgen del Rocio University Hospital/CSIC/University of Seville, 41013 Seville, Spain

**Keywords:** *Escherichia coli*, *Klebsiella pneumoniae*, urinary tract infection, urine pH, ciprofloxacin, fosfomycin, experimental murine model

## Abstract

In vitro studies have suggested that acidic pH may reduce and increase the efficacy of ciprofloxacin and fosfomycin, respectively, when used to treat *Escherichia coli* and *Klebsiella pneumoniae* infections. We assessed the effects of acidic, neutral, and alkaline urine pH on the efficacy of optimized ciprofloxacin and fosfomycin dosages in UTI murine model of *E. coli* and *K. pneumoniae*. Immunocompetent and immunocompromised mice with adjusted urine pH were inoculated with *E. coli* and *K. pneumoniae* strains, and the efficacy was assessed based on the bacterial concentrations in tissues and fluids at 72 h, with respect to untreated controls. At acidic urine pH, both antimicrobials were effective, achieving similar reductions in *E. coli* concentrations in the kidneys in immunocompetent and immunocompromised mice and in *K. pneumoniae* in immunocompetent mice. At a neutral urine pH, both therapies reduced the presence of *E. coli* in the kidneys of immunocompetent mice. However, in immunocompromised mice, antimicrobials were ineffective at treating *E. coli* infection in the kidneys at a neutral urine pH and showed reduced efficacy against *K. pneumoniae* at both acidic and neutral urine pH. The results showed no correlation between urine pH and antimicrobial efficacy, suggesting that the reduced effectiveness is associated with the animals’ immunocompetence status.

## 1. Introduction

Urinary tract infections (UTIs) are highly prevalent among kidney transplant recipients (KTRs), representing nearly half of all infectious complications in this population [1]. As is the case in the general population, *Escherichia coli* is the most frequent cause of UTI in KTRs [1,2], followed by *Klebsiella pneumoniae* [3]. Antimicrobial resistance among Gram-negative bacilli (GNB) has increased during the last decade, and this may be associated with risk factors commonly observed in the transplant setting [1,4,5]. Effective selection of antimicrobial agents is crucial for resolving these infections and mitigating the emergence of bacterial resistance [6]. The antibiotics most frequently used to treat Enterobacterales infections include β-lactams, β-lactams/β-lactamase inhibitors, fluoroquinolones, fosfomycin, and aminoglycosides, among others [7]. A recent study carried out in six European countries highlighted the highest resistance rates for antibiotics recommended for *E. coli* UTI treatment: 22.4% for trimethoprim/sulfamethoxazole, 16.7% for amoxicillin/clavulanic acid, and 15.1% for ciprofloxacin [8].

Ciprofloxacin is a second-generation fluoroquinolone antibiotic that interacts with DNA gyrase to influence transcription and DNA replication [9,10]. Its excellent bioavailability and high concentration in urine make it a good option for ambulatory management of UTIs [11,12]. However, in November 2018, the European Medicines Agency published a review detailing various serious, disabling, and potentially permanent side effects of fluoroquinolone antibiotics and recommended that they should not be used to treat bacterial infections in patients with kidney disease or in organ transplant recipients [13].

Fosfomycin, an antibiotic that is often prescribed to treat uncomplicated lower UTIs, has garnered attention due to its favorable pharmacokinetic and pharmacodynamics properties, including its high concentrations in urine [14,15]. Although *E. coli*’s resistance to fosfomycin remains relatively low (typically less than 3% [16]), recent reports indicate a concerning trend of rising resistance rates [17]. UTIs caused by extended-spectrum β-lactamase-producing Enterobacterales, such as *E. coli*, have similarly poor resistance to fosfomycin. Fosfomycin also exhibits activity against *K. pneumoniae*, with a susceptibility rate of 62% [18]. These findings underscore its potential as a valuable treatment for UTIs.

Information regarding the use of fosfomycin in immunocompromised patients, such as KTRs, is scarce. To our knowledge, thus far, only Rivera-Sánchez et al. have described fosfomycin as one of the main therapeutic options for treating UTIs in KTRs caused by ciprofloxacin-resistant Gram-negative isolates [19]. As such, there is a pressing need to assess alternative treatment options and understand the factors that affect antibiotic efficacy in the urinary tract microenvironment.

The microenvironment of the urinary tract may significantly influence the efficacy of antimicrobial agents [20,21,22,23]. While in vitro investigations have suggested that acidic pH or anaerobic conditions may adversely affect the bactericidal activity of ciprofloxacin [20,24], fosfomycin has demonstrated increased activity under such conditions [25]. However, there remains a notable gap in our understanding, as few in vivo studies have explored the impact of urine pH on the effectiveness of antibiotics in treating GNB UTIs [26,27]. In a recent observational study carried out in KTRs prescribed fosfomycin or ciprofloxacin therapy to treat UTI episodes caused by *E. coli* and *K. pneumoniae*, acidic urine pH was associated with symptomatic UTI episodes at one-month follow-up; this was particularly noticeable in patients who received fosfomycin therapy for *E. coli* episodes. Furthermore, at acidic pH, the minimum inhibitory concentration of ciprofloxacin against 90% of strains (MIC_90_) increased from 8 to >8 mg/L in *E. coli* and from 4 to >8 mg/L in *K. pneumoniae*, whereas the fosfomycin MIC_90_ decreased from 8 to 4 mg/L in *E. coli* and from 512 to 128 mg/L in *K. pneumoniae* [26]. In addition, acidic pH conditions increased the cells invasion by *E. coli* in human renal cell cultures, and such conditions were associated with enhanced bacterial concentrations and pyelonephritis-like symptoms in the kidneys in a lower UTI murine model of *E. coli* and *K. pneumoniae* infection [28].

To better characterize the association between different urine pHs, especially the acidic condition, and the antimicrobial efficacy, in the present study, we evaluated treatment with ciprofloxacin and fosfomycin in both immunocompetent and immunocompromised mice with *E. coli* or *K. pneumoniae* infections in their lower urinary tracts, considering the influence of different urinary pH conditions. By elucidating the impact of urinary pH on antibiotic efficacy, we aimed to provide valuable insights that will help to optimize strategies for treating UTIs.

## 2. Results

### 2.1. In Vitro Assays

#### 2.1.1. Time–Kill Curve Assays

**Figure 1:**
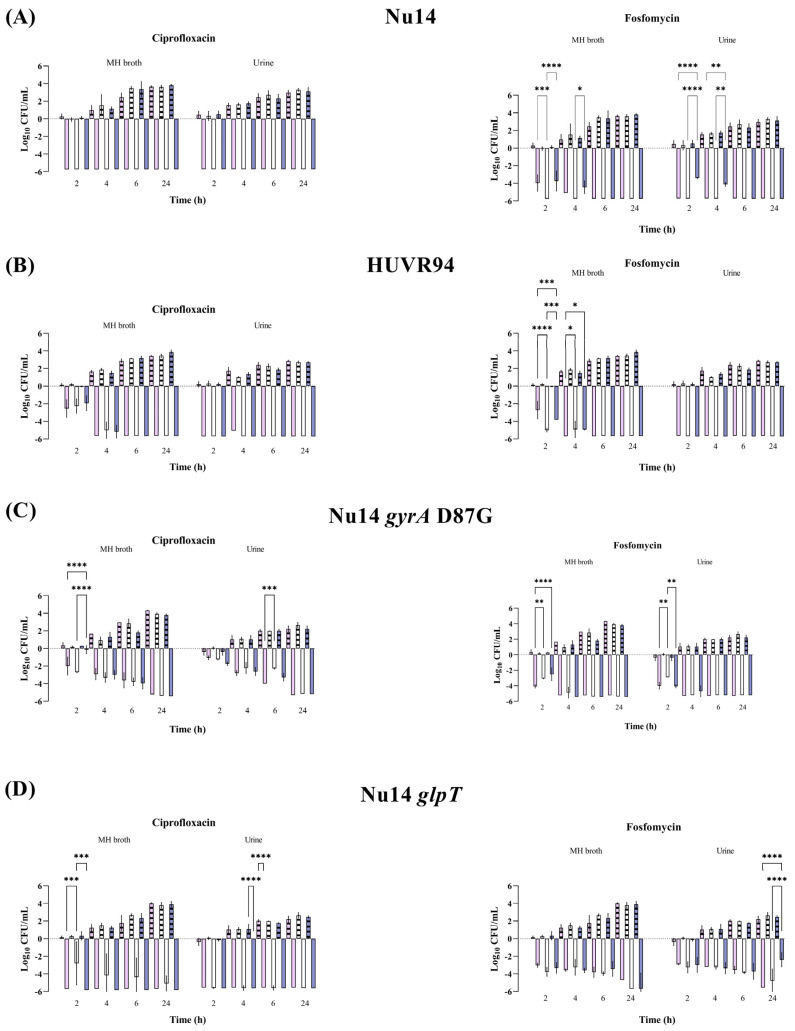
Bactericidal activity of ciprofloxacin and fosfomycin at C_max_ concentrations in MHB and urine, determined at pH 5 (pink bars), pH 7 (white bars), and pH 8 (purple bars), against *Escherichia coli* Nu14 (**A**), HUVR94 (**B**), Nu14 *gyrA* (**C**), and Nu14 *glpT* (**D**) strains. Results are represented as differences (log_10_ CFU/mL) relative to the initial timepoint (0 h). Striped bar: growth control; empty bar: antimicrobial condition at the C_max_ levels reached in C57BL/6J mice plasma (13.22 mg/L for ciprofloxacin and 1354.09 mg/L for fosfomycin). Differences in bacterial concentrations across pH levels at the same time point were compared using analysis of variance (ANOVA) followed by Dunnett’s and Tukey’s post hoc tests. Significant differences were observed between the antimicrobial condition and its corresponding growth control at all time points; however, the figure highlights only the statistically significant differences in pH within each time point. *: *p* < 0.05; **: *p* < 0.01; ***: *p* < 0.001; ****: *p* < 0.0001. Growth control curves for each strain and pH condition are represented in Supplementary Figure S1.

**Figure 2:**
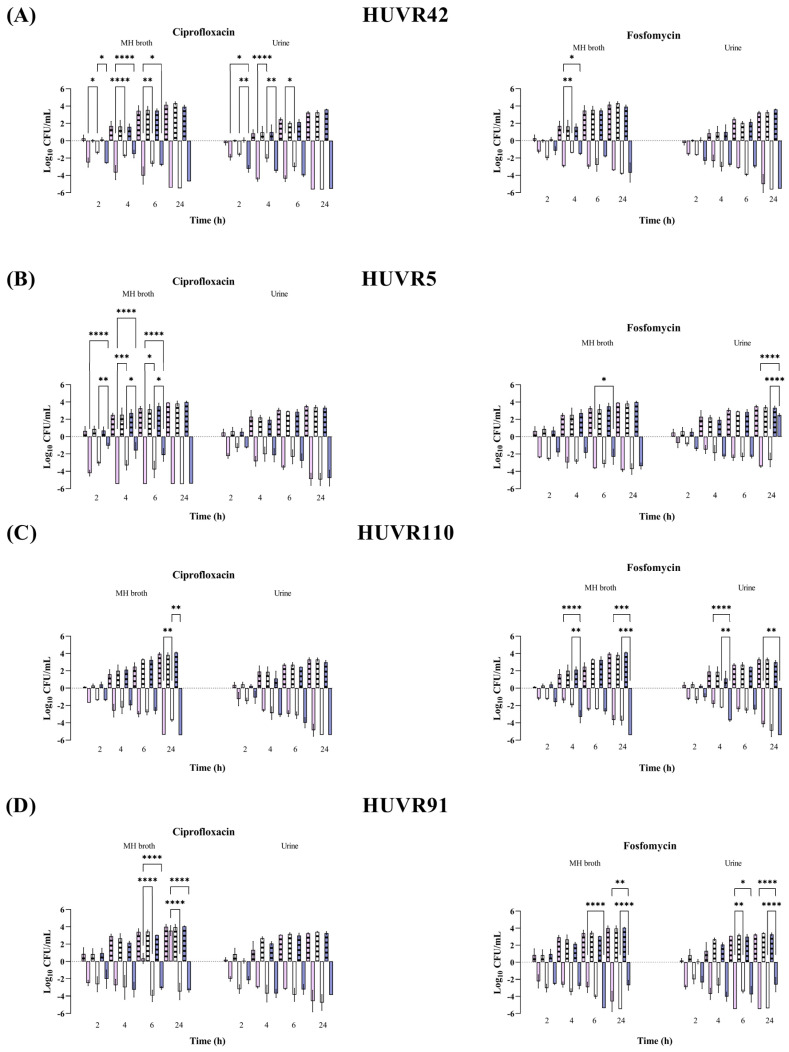
Bactericidal activity of ciprofloxacin and fosfomycin at C_max_ concentrations in MHB and urine, determined at pH 5 (pink bars), pH 7 (white bars), and pH 8 (purple bars), against *Klebsiella pneumoniae* HUVR42 (**A**), HUVR5 (**B**), HUVR110 (**C**), and HUVR91 (**D**) strains. Results are represented as differences (log_10_ CFU/mL) relative to the initial timepoint (0 h). Striped bar: growth control; empty bar: antimicrobial condition at the C_max_ levels reached in C57BL/6J mice plasma (13.22 mg/L for ciprofloxacin and 1354.09 mg/L for fosfomycin). Differences in bacterial concentrations across pH levels at the same time point were compared using analysis of variance (ANOVA) followed by Dunnett’s and Tukey’s post hoc tests. Significant differences were observed between the antimicrobial condition and its corresponding growth control at all time points; however, the figure highlights only the statistically significant differences in pH within each time point. *: *p* < 0.05; **: *p* < 0.01; ***: *p* < 0.001; ****: *p* < 0.0001. Growth control curves for each strain and pH condition are represented in Supplementary Figure S2.

At maximum serum concentration (mice C_max_, Supplementary Table S1), both antibiotics showed bactericidal activity against all *E. coli* strains and *K. pneumoniae* strains from 2 to 24 h and from 6 to 24 h, respectively; these effects were independent of the pH and growth media (Müller–Hinton Broth (MHB) or urine) (Figure 1 and Figure 2 and Supplementary Figures S1 and S2). No differences were observed in strain control growths between MHB or urine across the different pH conditions, although at 24 h, they were approximately 1 log_10_ CFU/mL lower in the urine experiments than in MHB. Among the *K. pneumoniae* strains, HUVR5 (which is resistant to both antimicrobials) showed regrowth in the fosfomycin assay at alkaline broth pH at 24 h, while HUVR91 (which is resistant to ciprofloxacin) exhibited regrowth in the ciprofloxacin assay from 6 to 24 h at acidic broth pH.

#### 2.1.2. Pharmacodynamics of Ciprofloxacin and Fosfomycin

The pharmacokinetics data, namely C_max_, t_1/2_, and AUC_0–24_, of ciprofloxacin and fosfomycin and their pharmacodynamics variables, namely AUC_0–24_/MIC, C_max_/MIC, and Δt/MIC, for the *E. coli* and *K. pneumoniae* strains are listed in Supplementary Table S1.

### 2.2. In Vivo Assays

#### 2.2.1. Impact of Acidic Urine pH and Immunocompromise on the Bacterial Concentrations in the Kidneys in a Lower UTI Model of *E. coli*

In immunocompetent and immunocompromised, untreated control mice infected with the four *E. coli* strains, the bacterial concentrations in the kidneys after 72 h were greater in the groups with acidic urine pH than in those with a neutral urine pH in a range from +1.75 to +2.19 and from +3.41 to +5.51 log_10_ CFU/g, respectively, and in the Nu14 strain with alkaline urine pH in immunocompromised mice (+3.11 log_10_ CFU/g; Supplementary Figures S3 and S4).

The immunocompromised, untreated control mice had higher bacterial concentrations in the kidneys at acidic urine pH than the immunocompetent mice infected with the *E. coli* Nu 14 and HUVR 94 strains (+2.2 and +2.25 log_10_ CFU/g, respectively). Conversely, at a neutral urine pH, the bacterial concentrations in the kidneys were lower in immunocompromised than in immunocompetent mice infected with the *E. coli* Nu14 *gyrA* D87G and Nu14 *glpT* missense mutation strains (−2.99 and −2.59 log_10_ CFU/g, respectively). There were no differences in the bacterial concentrations in the kidneys with regard to the immunocompetence at alkaline urine pH or in the blood stream infection (BSI) and mortality rates at any pH condition (Supplementary Tables S2–S4).

#### 2.2.2. Efficacy of Ciprofloxacin and Fosfomycin in an Immunocompetent Lower UTI Model of *E. coli*

**Figure 3:**
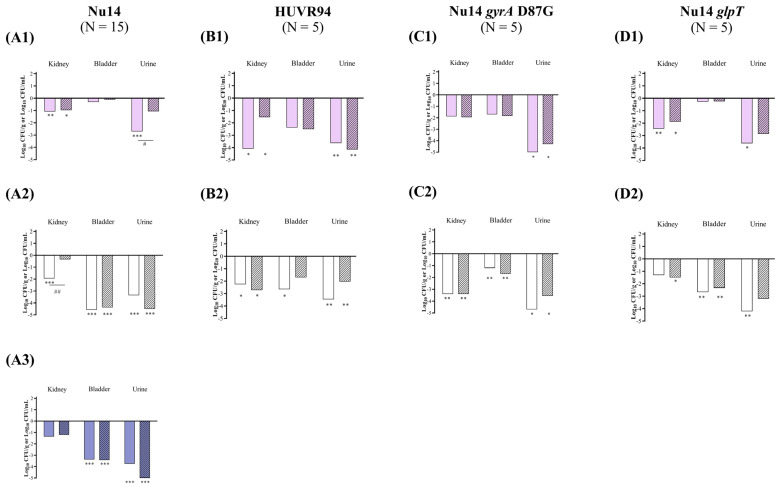
In vivo efficacy of ciprofloxacin and fosfomycin for the experimental urinary tract infection in immunocompetent mice model by *E. coli* Nu14 (**A**), HUVR94 (**B**), Nu14 *gyrA* (**C**), and Nu14 *glpT* (**D**) strains at acidic (1), neutral (2), and alkaline (3) urine pH. Ciprofloxacin− and fosfomycin−treated groups are represented as difference with respect to mean of bacterial concentration in control mice groups (log_10_ CFU/mL; Supplementary Figure S3). Empty bar: ciprofloxacin−treated mice; striped bar: fosfomycin−treated mice. The Mann–Whitney U test was used to compare quantitative variables. *: *p* < 0.05 compared to their control mice group; **: *p* < 0.01 compared to their control mice group; ***: *p* < 0.001 compared to their control mice group; ^#^: *p* < 0.05 compared to fosfomycin−treated mice group; ^##^: *p* < 0.01 compared to fosfomycin−treated mice group.

At acidic urine pH, ciprofloxacin reduced the bacterial concentrations of *E. coli* Nu14, HUVR94, and Nu14 *glpT* strains in the kidneys by −1.08, −4.08, and −2.44 log_10_ CFU/g, respectively, whereas fosfomycin reduced the bacterial concentrations of *E. coli* Nu14, HUVR94, and Nu14 *glpT* strains in the kidneys by −0.97, −1.54, and −1.88 log_10_ CFU/g, respectively. Ciprofloxacin reduced the bacterial concentrations of *E. coli* Nu14, HUVR94, Nu14 *gyrA*, and Nu14 *glpT* strains in the urine by −2.70, −3.63, −5.05, and −3.62 log_10_ CFU/mL, respectively, compared with untreated controls, while fosfomycin also reduced the concentrations of *E. coli* HUVR94 and Nu14 *gyrA* in the urine by −4.15 and −4.28 log_10_ CFU/mL, respectively (Figure 3 and Supplementary Figure S3). Ultimately, ciprofloxacin was more effective than fosfomycin when it came to reducing the bacterial concentration of *E. coli* Nu14 (−1.64 log_10_ CFU/mL) in urine. No differences were observed in the BSI and mortality rates in the control groups after both treatments (Supplementary Figure S5).

At neutral urine pH, ciprofloxacin decreased the bacterial concentrations of *E. coli* Nu14, HUVR94, and Nu14 *gyrA* strains in the kidneys by −1.94, −2.25, and −3.39 log_10_ CFU/g, respectively, and fosfomycin reduced the concentrations of *E. coli* HUVR94, Nu14 *gyrA*, and Nu14 *glpT* strains by −2.72, −3.39, and −1.50 log_10_ CFU/g, respectively. Moreover, ciprofloxacin outperformed fosfomycin in reducing the bacterial concentration in the kidneys of mice infected with *E. coli* Nu14 (−1.60 log_10_ CFU/g). Ciprofloxacin also reduced the bacterial concentrations of *E. coli* Nu14, HUVR94, Nu14 *gyrA*, and Nu14 *glpT* strains in the bladder by −4.57, −2.65, −1.21, and −2.67 log_10_ CFU/g, respectively, and it reduced the concentrations of *E. coli* strains in the urine by −3.35, −3.46, −4.69, and −4.21 log_10_ CFU/mL, respectively, compared with untreated controls. Fosfomycin reduced the bacterial concentrations of *E. coli* Nu14, Nu14 *gyrA*, and Nu14 *glpT* strains in the bladder by −4.39, −1.69, and −2.34 log_10_ CFU/g, respectively, compared to untreated controls, and it reduced *E. coli* Nu14 and Nu14 *gyrA* strains in the bladder by −4.66 and −3.55 log_10_ CFU/mL (Figure 3 and Supplementary Figure S3).

At alkaline pH, in mice infected with *E. coli* Nu14, both antibiotics effectively decreased the bacterial concentrations in the bladder and urine compared with those of the untreated control groups, from −3.37 to −3.42 log_10_ CFU/g and from −3.74 to −5.23 log_10_ CFU/mL, respectively (Figure 3 and Supplementary Figure S3). The BSI and mortality rates were 0% in both the untreated control and the ciprofloxacin− or fosfomycin−treated groups infected with *E. coli* Nu14 at alkaline pH.

#### 2.2.3. Efficacy of Ciprofloxacin and Fosfomycin in an Immunocompromised Lower UTI Model of E. coli

**Figure 4:**
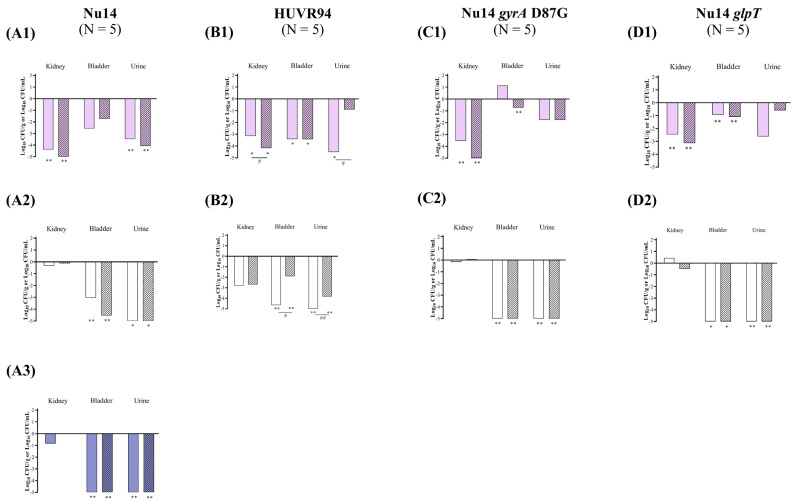
In vivo efficacy of ciprofloxacin and fosfomycin for the experimental urinary tract infection in immunocompromised mice model by *E. coli* Nu14 (**A**), HUVR94 (**B**), Nu14 *gyrA* (**C**), and Nu14 *glpT* (**D**) strains at acidic (1), neutral (2), and alkaline (3) urine pH. Ciprofloxacin− and fosfomycin−treated groups are represented as difference with respect to mean bacterial concentration of control mice group (log_10_ CFU/mL; Supplementary Figure S4). Empty bar: ciprofloxacin−treated mice; striped bar: fosfomycin−treated mice. The Mann–Whitney U test was used to compare quantitative variables. *: *p* < 0.05 compared to their control mice group; **: *p* < 0.01 compared to their control mice group; ^#^: *p* < 0.05 compared to fosfomycin−treated mice group; ^##^: *p* < 0.01 compared to fosfomycin−treated mice group.

At acidic urine pH, ciprofloxacin significantly reduced the bacterial concentrations of *E. coli* Nu14, HUVR94, Nu14 *gyrA*, and Nu14 *glpT* strains in the kidneys by −4.37, −3.15, −3.54, and −2.46 log_10_ CFU/g, respectively, while fosfomycin reduced the presence of *E. coli* Nu14, HUVR94, Nu14 *gyrA*, and Nu14 *glpT* strains in the kidneys by −5.58, −4.17, −5.49, and −3.13 log_10_ CFU/g, respectively. In immunocompromised mice, ciprofloxacin was less effective than fosfomycin at reducing the bacterial concentrations of *E. coli* HUVR94 in the kidneys, achieving a reduction of −1.02 log_10_ CFU/g. Ciprofloxacin also reduced the bacterial concentration of *E. coli* HUVR94 and Nu14 *glpT* in the bladder by −3.41 and −0.93 log_10_ CFU/g, respectively, and reduced the concentrations of *E. coli* Nu14 and HUVR94 strains in the urine by −3.47 and −4.51 log_10_ CFU/mL, respectively, compared with untreated controls. Fosfomycin reduced the concentrations of HUVR94, Nu14 *gyrA*, and Nu14 *glpT* in the bladder by −3.43, −0.72, and −1.08 log_10_ CFU/g, respectively, and reduced the bacterial concentration of Nu14 in the urine by −4.07 log_10_ CFU/mL (Figure 4 and Supplementary Figure S4). In accordance with the results of the immunocompetent mice, ciprofloxacin demonstrated better efficacy than fosfomycin in reducing the bacterial concentration of *E. coli* HUVR94 in the urine (−3.61 log_10_ CFU/mL). No differences were observed in the BSI and mortality rates after both treatments with respect to the control groups (Supplementary Figure S5).

At neutral urine pH, both antimicrobials were ineffective at reducing bacterial concentrations in the kidneys compared with untreated controls, which exhibited low bacterial concentrations in the kidneys, with means from 0.66 to 3.75 log_10_ CFU/g. However, both ciprofloxacin and fosfomycin effectively reduced the bacterial concentrations of the four *E. coli* strains in the bladder and the urine. Thus, ciprofloxacin reduced the bacterial concentrations of Nu14, HUVR94, Nu14 *gyrA*, and Nu14 *glpT* strains in the bladder by −3.03, −4.65, −5.26, and −6.75 log_10_ CFU/g, respectively, while fosfomycin reduced the concentrations by −4.54, −1.90, −6.60, and −6.46 log_10_ CFU/g, respectively. Additionally, ciprofloxacin decreased the bacterial concentrations of Nu14, HUVR94, Nu14 *gyrA*, and Nu14 *glpT* in the urine by −6.37, −8.74, −7.71, and −8.37 log_10_ CFU/mL, respectively, while fosfomycin reduced their concentrations by −5.52, −3.83, −6.14, and −6.19 log_10_ CFU/mL. Ciprofloxacin also outperformed fosfomycin in terms of bladder and urine clearance in mice infected with *E. coli* HUVR94 (−2.75 log_10_ CFU/g and −3.21 log_10_ CFU/mL, respectively).

At alkaline pH, in mice infected with *E. coli* Nu14, similar to the findings in the immunocompetent mice, both antimicrobials reduced the bacterial concentrations in the bladder (−5.41 and −5.31 log_10_ CFU/g) and urine (−5.37 and −5.28 log_10_ CFU/mL) with respect to those of the untreated mice (Figure 4 and Supplementary Figure S4). Additionally, the BSI and mortality rates were 0% for both the untreated control and the ciprofloxacin− or fosfomycin−treated groups, although BSI was observed in one out of the five mice in the ciprofloxacin−treated group.

#### 2.2.4. Impact of Acidic Urine pH and Immunocompromise on the Bacterial Concentrations in the Kidneys in a Lower UTI Model of *K. pneumoniae*

In immunocompetent and immunocompromised, untreated control mice infected with *K. pneumoniae*, the bacterial concentrations in the kidneys after 72 h were greater in the groups with acidic pH urine than in those with neutral urine pH, in a range from +1.90 to +4.43 and from +3.95 to +4.28 log_10_ CFU/g, and in the HUVR42 strain group with alkaline urine pH from +3.48 to +4.84 log_10_ CFU/g (Supplementary Figures S7 and S8).

There were no differences in the bacterial concentrations in the kidneys at acidic and neutral urine pH when we compared the immunocompromised and immunocompetent, untreated control mice infected with *K. pneumoniae* strains. Only the HUVR42 strain differed, showing a higher bacterial concentration in the kidneys of immunocompromised mice (+1.64 log_10_ CFU/g) at neutral urine pH condition. In addition, at acidic urine pH, HUVR91 produced 100% BSI in immunocompromised mice vs. 0% in the immunocompetent mice. Immunocompetence did not affect the mortality rates of the mice at any pH condition (Supplementary Tables S2–S4).

#### 2.2.5. Efficacy of Ciprofloxacin and Fosfomycin in an Immunocompetent Lower UTI Model of *K. pneumoniae*

**Figure 5:**
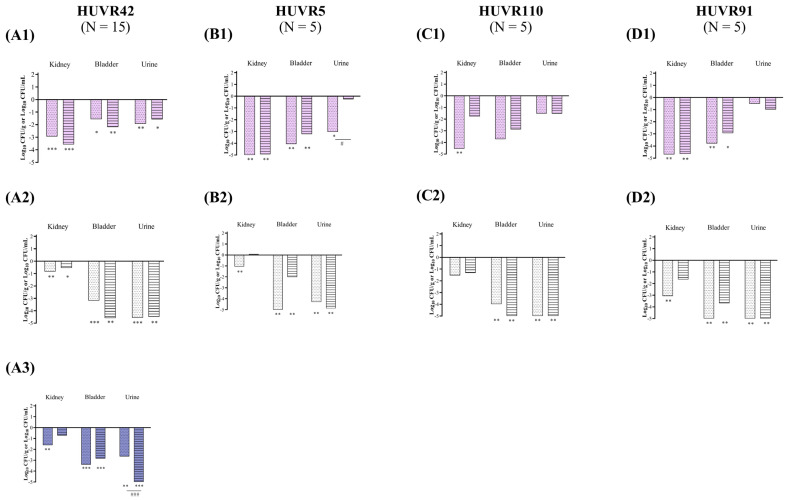
In vivo efficacy of ciprofloxacin and fosfomycin for the experimental urinary tract infection in immunocompetent mice model by *K. pneumoniae* HUVR42 (**A**), HUVR5 (**B**), HUVR110 (**C**), and HUVR91 (**D**) strains at acidic (1), neutral (2), and alkaline (3) urine pH. Ciprofloxacin− and fosfomycin−treated groups are represented as difference with respect to mean bacterial concentration of control mice group (log_10_ CFU/mL; Supplementary Figure S7). Empty bar: ciprofloxacin−treated mice; striped bar: fosfomycin−treated mice. The Mann–Whitney U test was used to compare quantitative variables. *: *p* < 0.05 compared to their control mice group; **: *p* < 0.01 compared to their control mice group; ***: *p* < 0.001 compared to their control mice group; ^#^: *p* < 0.05 compared to fosfomycin−treated mice group; ^###^: *p* < 0.001 compared to fosfomycin−treated mice group.

At acidic urine pH, compared with untreated controls, ciprofloxacin reduced the bacterial concentrations of *K. pneumoniae* HUVR42, HUVR5, HUVR110, and HUVR91 strains in the kidneys by −2.94, −5.86, −4.54, and −4.69 log_10_ CFU/g, respectively, while fosfomycin reduced the concentrations of *K. pneumoniae* HUVR42, HUVR5, and HUVR91 by −3.57, −4.93, and −4.65 log_10_ CFU/g, respectively. Ciprofloxacin reduced the bacterial concentrations of HUVR42, HUVR5, and HUVR91 in the bladder by −1.57, −4.07, and −3.79 log_10_ CFU/g, respectively, and reduced the concentrations of HUVR42 and HUVR5 strains in the urine by −1.93 and −3.03 log_10_ CFU/mL, respectively. Fosfomycin reduced the bacterial concentrations of HUVR42, HUVR5, and HUVR91 in the urine by −2.20, −3.23, and −2.93 log_10_ CFU/g, respectively, and reduced the concentrations of *K. pneumoniae* HUVR42 in the urine by −1.58 log_10_ CFU/g (Figure 5 and Supplementary Figure S7). Additionally, ciprofloxacin showed superior efficacy to fosfomycin when it came to reducing the bacterial concentration of *K. pneumoniae* HUVR5 in the urine (−2.77 log_10_ CFU/mL). No differences were observed in the BSI and mortality rates after both treatments with respect to the control groups (Supplementary Figure S5).

At neutral urine pH, ciprofloxacin reduced the bacterial concentrations of *K. pneumoniae* HUVR42 and HUVR91 in the kidneys by −0.84 and −3.12 log_10_ CFU/g, while fosfomycin achieved a similar reduction for *K. pneumoniae* HUVR42 alone (−0.55 log_10_ CFU/g). Both antimicrobials significantly decreased the bacterial concentrations of *K. pneumoniae* HUVR42, HUVR5, HUVR110, and HUVR91 in the bladder and in the urine. Ciprofloxacin reduced the bacterial concentrations in the bladder by −3.18, −5.01, −3.98, and −5.57 log_10_ CFU/g, respectively, and reduced the urine bacterial concentrations of the same four *K. pneumoniae* strains by −4.56, −4.29, −5.90, and −8.04 log_10_ CFU/mL, respectively, compared with untreated controls. Fosfomycin reduced the bacterial concentrations of HUVR42, HUVR5, HUVR110, and HUVR91 in the bladder by −4.59, −2.02, −5.66, and −3.69 log_10_ CFU/g, respectively, and in the bladder, it reduced the concentrations of the same four *K. pneumoniae* strains by −4.48, −4.89, −6.83, and −8.44 log_10_ CFU/mL, respectively, compared with untreated controls. Moreover, ciprofloxacin outperformed fosfomycin in reducing the bacterial concentration of *K. pneumoniae* HUVR5 in the bladders of infected mice (−2.99 log_10_ CFU/g).

At alkaline pH, ciprofloxacin reduced the bacterial concentration of HUVR42 in the kidneys with respect to the concentrations observed in untreated controls (−1.62 log_10_ CFU/g). Both antibiotics decreased the bacterial concentrations in the bladder (−2.85 to −3.40 log_10_ CFU/g) and urine (−2.65 to −5.94 log_10_ CFU/mL) compared with the concentrations observed in the untreated control groups. However, fosfomycin was more effective than ciprofloxacin at reducing the bacterial concentrations in urine (−3.29 log_10_ CFU/mL) (Figure 5 and Supplementary Figure S7). The BSI and mortality rates were 0% in the untreated control and ciprofloxacin- or fosfomycin-treated groups infected with *K. pneumoniae* HUVR42 at alkaline pH, though BSI was observed in two out of fifteen mice in the untreated control group.

#### 2.2.6. Efficacy of Ciprofloxacin and Fosfomycin in an Immunocompromised Lower UTI Model of *K. pneumoniae*

**Figure 6:**
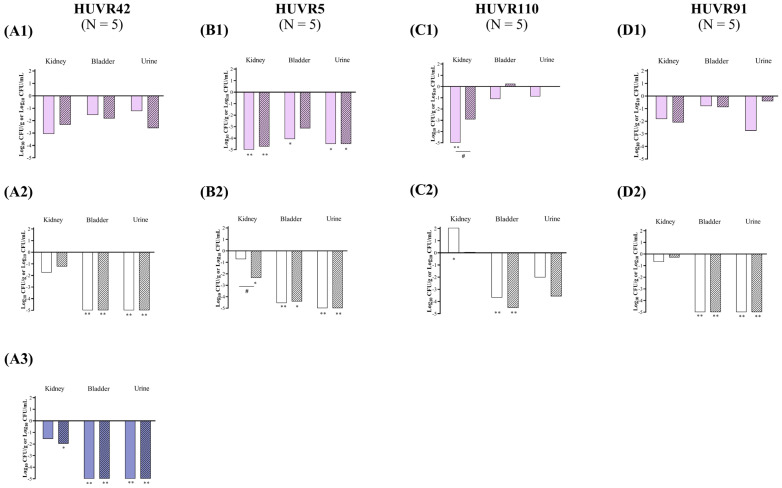
In vivo efficacy of ciprofloxacin and fosfomycin for the experimental urinary tract infection in immunocompromised mice model by *K. pneumoniae* HUVR42 (**A**), HUVR5 (**B**), HUVR110 (**C**), and HUVR91 (**D**) strains at acidic (1), neutral (2), and alkaline (3) urine pH. Ciprofloxacin− and fosfomycin−treated groups are represented as difference with respect to mean bacterial concentration of control mice group (log_10_ CFU/mL; Supplementary Figure S8). Empty bar: ciprofloxacin−treated mice; striped bar: fosfomycin−treated mice. The Mann–Whitney U test was used to compare quantitative variables. *: *p* < 0.05 compared to their control mice group; **: *p* < 0.01 compared to their control mice group; ^#^: *p* < 0.05 compared to fosfomycin−treated mice group.

At acidic urine pH, ciprofloxacin reduced the bacterial concentrations of *K. pneumoniae* HUVR5 and HUVR110 strains in the kidneys by −5.29 and −5.17 log_10_ CFU/g, respectively, and reduced the concentrations of HUVR5 in the bladder and urine by −4.49 log_10_ CFU/g and −4.07 log_10_ CFU/mL, respectively, compared with untreated controls. However, fosfomycin reduced the bacterial concentrations of *K. pneumoniae* HUVR5 in the kidneys (−4.72 log_10_ CFU/g), bladder (−3.14 log_10_ CFU/g), and urine (−4.49 log_10_ CFU/mL) (Figure 6 and Supplementary Figure S8). No differences were observed in the BSI and mortality rates after both treatments with respect to the control groups (Supplementary Figure S5).

At neutral urine pH, fosfomycin reduced the bacterial concentrations of *K. pneumoniae* HUVR5 in the kidneys (−2.35 log_10_ CFU/g). Both antimicrobials reduced the bacterial concentrations in the bladder and urine for all four studied strains, while the concentrations were similarly reduced for three strains in the untreated controls. Ciprofloxacin reduced the bacterial concentrations of *K. pneumoniae* HUVR42, HUVR5, HUVR110, and HUVR91 in the bladder by −6.59, −4.55, −3.68, and −6.25 log_10_ CFU/g, respectively, while in the urine, it reduced the concentrations of HUVR42, HUVR5, and HUVR91 by −7.53, −5.66, and −5.22 log_10_ CFU/mL, respectively. Fosfomycin proved to be an effective treatment for HUVR42, HUVR5, HUVR110, and HUVR91, reducing their concentrations by −6.18, −4.41, −4.53, and −5.02 log_10_ CFU/g, respectively, while in urine, it was effective for HUVR42, HUVR5, and HUVR91, reducing them by −7.67, −6.36, and −5.72 log_10_ CFU/mL.

Finally, at alkaline pH, fosfomycin significantly reduced the kidneys’ bacterial concentrations with respect to untreated mice infected with *K. pneumoniae* HUVR42 by −1.96 log_10_ CFU/g. Ciprofloxacin and fosfomycin also decreased the bacterial concentrations in the bladder (−5.00 and −5.09 log_10_ CFU/g) and urine (−7.18 and −7.98 log_10_ CFU/mL) with respect to the untreated controls (Figure 6 and Supplementary Figure S8). The BSI rates were 60%, 40%, and 0% in the untreated control, ciprofloxacin, and fosfomycin groups. No mortality was observed at alkaline urine pH in mice infected with the *K. pneumoniae* HUVR42 strain.

## 3. Discussion

At acidic urine pH, ciprofloxacin and fosfomycin demonstrated similar efficacy in reducing the bacterial concentrations in the kidneys in both immunocompetent and immunocompromised mice with *E. coli* in their lower urinary tracts in spite of the higher bacterial concentrations found in the kidneys in the untreated control groups at the end of the 72 h experimental period. Moreover, both antimicrobials successfully reduced kidney infection at neutral urine pH in the immunocompetent mouse model. Notably, both antimicrobials were ineffective at reducing kidney infection in immunocompromised mice at neutral and at alkaline urine pH.

Regarding the *K. pneumoniae* lower UTI model in the immunocompetent mice, both ciprofloxacin and fosfomycin effectively reduced the bacterial concentrations in the kidneys for most of the strains at acidic urine pH, successfully treating two of the four strains, but they showed reduced efficacy at neutral urine pH, at which only one strain was affected; moreover, ciprofloxacin achieved a greater reduction in the bacterial concentrations in the kidneys with regard to the strain tested at alkaline urine pH. In the immunocompromised mouse model, both ciprofloxacin and fosfomycin showed reduced efficacy at acidic urine pH, successfully treating two and one of the four strains, respectively. At neutral and alkaline urine pH, only fosfomycin reduced the level of infection for one out of the four strains and for the unique tested strain, respectively. The antimicrobial efficacy was similar to the results of the bacterial eradication from the bladder and urine at any urine pH and immunocompetence and in the *E. coli* and *K. pneumoniae* lower UTI models, though small differences were observed for specific strains.

In accordance with results previously reported [20,24] for *E. coli* strains, time–kill curves showed that both antibiotics exhibited bactericidal activity against *E. coli* and *K. pneumoniae* strains at C_max_ concentrations independently of the pH and growth media. While no growth differences were noted between MHB or urine across pH conditions, the bacterial growth in urine was lower at 24 h compared to that in MHB, which was also consistent with previous reports [20,24]. Regarding the impact of pH of the medium on the in vitro antimicrobial activity, ciprofloxacin is less active at acidic pH and exhibits higher MIC concentrations [20,24], while fosfomycin remains marginally active independently of the media [26]. Martín-Gutiérrez et al. [25] observed a decrease in fosfomycin activity in MHB at alkaline pH in vitro, with an increase in the fosfomycin MIC against susceptible *E. coli* strains and strains with a low level of fosfomycin resistance. These findings are in agreement with previous studies suggesting that changes in media pH affect the activity of different antibiotics [29]. pH influences ciprofloxacin and fosfomycin activity, leading to changes in their molecular structure and ionization state [30]. The activity of ciprofloxacin, optimal at neutral pH, where it exists predominantly in zwitterionic and neutral forms, enhances bacterial permeability and absorption [31]. However, in acidic environments, typical in urine (pH ≤ 6.5) [32], ciprofloxacin is presented in its cationic form, reducing its ability to penetrate and decreasing its activity [33]. However, in the case of fosfomycin, the acidic environment enhances its activity, and being in an anaerobic environment also activates the expression of GlpT-binding and UhpT efflux pumps [34], increasing susceptibility to fosfomycin among uropathogenic bacteria. The antimicrobial activity of other antimicrobials also appears to be influenced by the pH. Thus, a recent in vitro study showed that a low pH reduces the activity of other antibiotics, such as ceftolozane/tazobactam and meropenem, against *E. coli* and *K. pneumoniae* in human urine medium [21]. These results were not completely reproduced in the present study when the assayed concentrations were increased to C_max_. At this concentration, scarce regrowth was observed during the experiments; interestingly, however, bacterial regrowth at 24 h after incubation with fosfomycin was observed at alkaline urine pH; likewise, bacterial regrowth occurred 24 h after incubation with ciprofloxacin at acidic urine pH, highlighting an interplay between antibiotic activity and urinary pH.

The in vivo efficacy of ciprofloxacin is associated with the AUC_0–24_/MIC pharmacodynamic (PD) parameter, and it has been reported to be static with a value of ~35 and with a maximal effect on values ≥ 100 [35]. In addition, data from an experimental pneumonia murine model infected with *K. pneumoniae* indicate that an AUC_0–24_/MIC ratio of >30 is bactericidal [36]. In the current study, the AUC_0–24_/MIC ratio for six of the *E. coli* and *K. pneumoniae* strains was >100; only two of them had ratios < 100 but higher than 30, without consistent differences in the efficacy of ciprofloxacin against the different strains, which supports the previously reported data. In addition, the volume of distribution of ciprofloxacin was approximately 3 L/kg, with tissues and urine concentrations higher than the serum levels [37]. In the case of fosfomycin, the results of neutropenic murine thigh models infected with *E. coli* and *K. pneumoniae* strains showed that the *f*AUC/MIC was the relevant PD parameter for these Gram-negative bacilli [38]. Based on these results, a population PD study in patients with *E. coli* bacteremia in the urinary tract showed that fosfomycin at a mean C_max_ between 400 and 500 mg/L—which was lower than that in our experimental model—had a 93.9% probability of achieving a bacteriostatic effect for an MIC of 128 mg/L and between 89% and 96% probability of decreasing the 1-log bacterial burden for an MIC of 32 mg/L [39]. Fosfomycin achieved high urine concentrations; thus, after an oral dose of 3 g, the serum C_max_ was 22–32 mg/L, and the urine concentration was 2000 mg/L [40]. Considering the higher C_max_ achieved with the dosage administered in our study, the results are consistent with the fosfomycin PK/PD data.

This study is the first to use both immunocompetent and immunocompromised mice to investigate the effects of urine pH on the efficacy of ciprofloxacin and fosfomycin as treatments for UTIs caused by *E. coli* and *K. pneumoniae* strains. The lack of efficacy of both antimicrobials in immunocompromised mice at neutral urine pH in the lower UTI model of *E. coli* and their lesser efficacies in the immunocompromised mice at acidic urine pH in the UTI model of *K. pneumoniae* are in line with the higher symptomatic risk of symptomatic UTI at one-month follow-up in KTRs with acidic urine pH [26]. Despite its optimal PK/PD parameters [14,15,41] and enhanced activity at acidic pH [25], fosfomycin exhibited similar effectiveness to ciprofloxacin when it came to reducing the bacterial concentrations in the tissues and urine of mice with UTIs caused by *E. coli* or *K. pneumoniae* strains across the three studied pH conditions.

Differences in the treatment responses between immunocompetent and immunocompromised mice shed light on the intricate dynamics between antimicrobial therapy, host immune function, and bacterial pathogenesis in UTIs. While both antimicrobials were effective at different urinary pH conditions in immunocompetent mice, divergent responses in immunocompromised mice are significant in the context of vulnerable patient populations such as KTRs. These individuals, who are often immunosuppressed to prevent graft rejection, face increased risks of severe or recurrent UTIs due to their compromised immune defenses [42,43,44]. Furthermore, the contraindication of ciprofloxacin in KTRs due to its potential for nephrotoxicity underscores the urgency of identifying alternative treatment options [13]. Fosfomycin is a promising candidate for this patient population. However, further research is needed to elucidate its long-term safety profiles.

The present study has several limitations. Firstly, the findings provide valuable insights into antimicrobial efficacy, but they are based on murine models, which may not fully mimic the complexities of human UTIs. Thus, we must be cautious when extrapolating the results to clinical practice. Additionally, potential challenges associated with chronic or recurrent infections were not considered. Furthermore, short-term mouse models are unsuitable for assessing whether immunocompromised models exhibit any renal function impairment after ciprofloxacin treatment, which has previously been observed in humans. Moreover, the lack of fosfomycin susceptibility breakpoints in Enterobacterales other than *E. coli* highlights the difficulties in interpreting susceptibility data and informing treatment decisions for UTIs caused by *K. pneumoniae*. These limitations emphasize the need to update clinical guidelines to optimize antimicrobial therapy. Finally, in spite of these limitations, the in vivo results did not confirm an association of acidic pH with lower or higher antimicrobial efficacy of ciprofloxacin and fosfomycin, respectively, as suggested by the previous in vitro activity studies, suggesting that the urine pH is not clinically important when an optimized antimicrobial dosage is prescribed.

## 4. Materials and Methods

### 4.1. Bacterial Strains

Experiments were conducted using four uropathogenic strains of both *E. coli* and *K. pneumoniae*, with the susceptibility/resistance profiles (MICs) of ciprofloxacin and fosfomycin previously characterized by broth microdilution and agar dilution methods, respectively, in accordance with the guidelines issued by the European Committee on Antimicrobial Susceptibility Testing [45]. The strains and their MICs of ciprofloxacin and fosfomycin, respectively, were (i) *E. coli* Nu14 wild type [46], 0.03 mg/L and 2 mg/L; (ii) *E. coli* HUVR94, 0.03 mg/L and 0.50 mg/L; (iii) *E. coli* Nu14 with a point mutation in *gyrA* D87G with LLQR [47], 0.25 mg/L and 0.50 mg/L, where *gyrA* encodes DNA gyrase, the target enzyme of ciprofloxacin, and mutations are associated with resistance to fluoroquinolones; (iv) *E. coli* Nu14 with a *glpT* missense mutation [48], 0.01 mg/L and 32 mg/L, where fosfomycin is transported into the cell via the GlpT and UhpT transporters, and defects in transport systems can confer fosfomycin resistance; (v) *K. pneumoniae* HUVR42, 0.007 mg/L and 4 mg/L; (vi) *K. pneumoniae* HUVR5, 8 mg/L and 128 mg/L; (vii) *K. pneumoniae* HUVR110, 8 mg/L and 4 mg/L; and (viii) *K. pneumoniae* HUVR91, 0.06 mg/L and 64 mg/L [26].

### 4.2. Time–Kill Curve Assays

Bactericidal time-dependent studies were carried out in triplicate in MHB (Thermo Scientific, Oxford, UK) and urine obtained from healthy volunteers who had not undergone antibiotic treatment in the previous three months. The urine samples were pooled and sterilized by filtration (using polyethersulfone membrane filters, 0.22 µm, VWR; Leicestershire, UK) and stored at 4 °C prior to analysis. For each strain, the bactericidal activity of ciprofloxacin and fosfomycin was assessed at concentrations equivalent to the C_max_ obtained in healthy mice with the doses used in the in vivo experiments: For ciprofloxacin, C_max_ was 13.22 mg/L [49], whereas for fosfomycin, it was 1354.09 mg/L [50]. The MHB and/or urine were adjusted to obtain acidic pH (pH = 5) or alkaline pH values (pH = 8) by adding 0.012% (*v*/*v*) of 12 N HCl or 0.072% (*v*/*v*) of 2 N NaOH, respectively (Sigma-Aldrich, Madrid, Spain). The initial inoculum was 5 × 10^5^ CFU/mL, and tubes were incubated at 37 °C with shaking; samples were taken at 0, 2, 4, 8, and 24 h and then serially diluted and seeded in 5% sheep blood plates. Bactericidal activity was defined as a decrease of ≥3 log_10_ CFU/mL from the initial inoculum [51].

### 4.3. In Vivo Assays

#### 4.3.1. Animals

Immunocompetent C57BL/6J female mice weighing 20 g (Production and Experimentation Animal Centre, US, Seville, Spain) were used in this study. Male mice were not included to avoid potential urogenital trauma that could occur during catheterization. The mice were murine pathogen-free, and their genetic authenticity was assessed. The animals were housed in individually ventilated cages under specific pathogen-free conditions, with water and food supplied ad libitum. This study was carried out in accordance with the recommendations of the Guide for the Care and Use of Laboratory Animals [52]. The experiments were approved by the Committee on the Ethics of Animal Experiments of *Consejería de Agricultura*, *Pesca y Desarrollo Rural*, Spain (06/03/2018/022), and *Consejería de Agricultura*, *Ganadería*, *Pesca y Desarrollo Sostenible* (16/11/2020/134). The sample size adhered to the 3Rs for the ethical use of animals in scientific research [53]. All procedures were performed under ketamine/xylazine anesthesia (Pfizer, Spain/Bayer Hispania S.L.; Madrid, Spain), and sodium thiopental was used for the sacrifices (B. Braun Medical S.A.; Barcelona, Spain).

#### 4.3.2. Efficacy of Ciprofloxacin and Fosfomycin Therapy in a Lower UTI Model with Different Urine pH in Immunocompetent and Immunocompromised Mice

A lower UTI murine model was previously characterized at different urine pHs [28]. Three days prior to inoculation, drinking water was replaced daily with (i) water containing 5% glucose for the neutral urine pH group; (ii) water containing 5% glucose and 0.5% NaHCO_3_ for the alkaline urine pH group [54]; and (iii) water containing 100 mM sucrose plus 0.56 M NH_4_Cl for the acidic urine pH group [55]. For the immunocompromised female C57BL/6J mouse model, the same protocol was followed when adjusting the urine pH, and 150 mg/kg/intraperitoneally (ip) and 100 mg/kg/ip of cyclophosphamide were administered 4 and 1 days prior to bacterial inoculation (day 0), respectively [56]. On the day of the inoculation, the mice were anesthetized with ketamine/xylazine (ip) and received transurethral inoculation with 50 μL of the previously characterized doses: 9 log_10_ CFU/mL for *E. coli* Nu14 and *E. coli* HUVR 94 and 8 log_10_ CFU/mL for all the other strains. The inoculum required to induce UTI in immunocompromised mice was one log_10_ CFU/mL fewer than that in wild-type mice [28]. Treatments were initiated 48 h post inoculation and lasted 24 h. Clinical formulations of ciprofloxacin (Spanish generics S.A.; Madrid, Spain) and fosfomycin (ERN; Madrid, Spain) were used. The animal weights and urine pH were recorded daily.

For both the immunocompetent and immunocompromised models and for the three conditions of urine pH, mice inoculated with each strain were randomly assigned to different groups (N = 5–15): (i) controls (untreated); (ii) ciprofloxacin, 20 mg/kg/12 h/ip; and (iii) fosfomycin, 500 mg/kg/8 h/ip. The antimicrobial dosages were determined based on pharmacokinetics and pharmacodynamics (PK/PD) data and their proven efficacy in previous murine models of infection [49,50]. Mortality was recorded for 24 h after the treatments began. Immediately after the animals’ death or when they were euthanized at the end of the experiment (sodium thiopental, ip), the kidneys, bladder, and urine were aseptically obtained and processed for quantitative cultures (log_10_ CFU/g and log_10_ CFU/mL). Blood samples were also recovered via cardiac puncture and studied for qualitative cultures.

### 4.4. Statistical Analysis

Differences in bacterial concentrations across pH levels at the same time point were compared using analysis of variance (ANOVA) followed by Dunnett’s and Tukey’s post hoc tests. Mortality and positive blood culture rates are expressed as percentages (%), while bacterial tissues and urine concentrations are presented as the mean ± standard deviation of log_10_ CFU/g and log_10_ CFU/mL, respectively. The chi-square or Fisher’s exact test was used to compare the mortality and BSI among groups. The Mann–Whitney U test was used to compare quantitative variables. A *p* < 0.05 was considered to be statistically significant. The statistical analysis was conducted using SPSS v24.0 software (SPSS Inc., Chicago, IL, USA).

## 5. Conclusions

Our results on the in vivo antimicrobial efficacy of ciprofloxacin and fosfomycin in murine lower UTI models do not support the in vitro data showing lower and higher activity of these antimicrobials, respectively, in media with acidic pH. In the lower UTI model at acidic urine pH, both ciprofloxacin and fosfomycin were similarly effective when reducing the bacterial concentrations of *E. coli* strains in the kidneys of both immunocompetent and immunocompromised mice and the concentrations of *K. pneumoniae* strains in kidneys of the immunocompetent mice. Moreover, at neutral urine pH, both antimicrobials effectively treated *E. coli* infections in the kidneys of the immunocompetent mice. However, the data suggest an association between reduced efficacy and immunocompromise. For immunocompromised mice infected with *E. coli*, both antimicrobials were ineffective at reducing infection in the kidneys at neutral urine pH; similarly, both ciprofloxacin and fosfomycin were less effective when treating *K. pneumoniae* at acidic and neutral urine pH.

## Data Availability

The data presented in this study are available on request from the corresponding authors.

## Supplementary Materials

The following supporting information can be downloaded at https://www.mdpi.com/article/10.3390/antibiotics13090827/s1, Supplementary Figure S1: Growth curves of *Escherichia coli* strains in MHB and urine, at pH 5 (pink), pH 7 (black), and pH 8 (purple); Supplementary Figure S2: Growth curves of *Klebsiella pneumoniae* strains in MHB and urine, at pH 5 (pink), pH 7 (black), and pH 8 (purple); Supplementary Figure S3: In vivo efficacy of ciprofloxacin and fosfomycin for the experimental urinary tract infection in immunocompetent mice model by four *Escherichia coli* strains at acidic (pink), neutral (white), and alkaline (purple) urine pH; Supplementary Figure S4: In vivo efficacy of ciprofloxacin and fosfomycin for the experimental urinary tract infection in immunocompromised mice model by four *Escherichia coli* strains at acidic (pink), neutral (white), and alkaline (purple) urine pH; Supplementary Figure S5: Bloodstream infection and mortality rates in lower urinary tract infection models by *Escherichia coli* and *Klebsiella pneumoniae* strains in immunocompetent and immunocompromised mice with acidic urine pH at 72 h of infection and treated with ciprofloxacin and fosfomycin; Supplementary Figure S6: Bloodstream infection and mortality rates in lower urinary tract infection models by *Escherichia coli* and *Klebsiella pneumoniae* strains in immunocompetent and immunocompromised mice with neutral urine pH at 72 h of infection and treated with ciprofloxacin and fosfomycin; Supplementary Figure S7: In vivo efficacy of ciprofloxacin and fosfomycin for the experimental urinary tract infection in immunocompetent mice model by four *Klebsiella pneumoniae* strains at acidic (pink), neutral (white), and alkaline (purple) urine pH; Supplementary Figure S8: In vivo efficacy of ciprofloxacin and fosfomycin for the experimental urinary tract infection in immunocompromised mice model by four *Klebsiella pneumoniae* strains at acidic (pink), neutral (white), and alkaline (purple) urine pH; Supplementary Table S1: Pharmacokinetics of ciprofloxacin and fosfomycin in healthy C57BL/6J female mice following administration of a single intraperitoneal dose and pharmacodynamics for *Escherichia coli* Nu14, HUVR94, *gyrA*, and *glpT* and *Klebsiella pneumoniae* HUVR42, HUVR5, HUVR110, and HUVR91 strains; Supplementary Table S2: Bacterial concentration in tissues and urine and BSI and mortality rates at acidic urine pH conditions in immunocompromised and immunocompetent mice after 72 h of lower urinary tract inoculation by *Escherichia coli* and *Klebsiella pneumoniae* strains; Supplementary Table S3: Bacterial concentration in tissues and urine and BSI and mortality rates at neutral urine pH conditions in immunocompromised and immunocompetent mice after 72 h of lower urinary tract inoculation by *Escherichia coli* and *Klebsiella pneumoniae* strains; Supplementary Table S4: Bacterial concentration in tissues and urine and BSI and mortality rates at alkaline urine pH condition in immunocompromised and immunocompetent mice after 72 h of lower urinary tract inoculation by *Escherichia coli* Nu14 and *Klebsiella pneumoniae* HUVR42 strains.
