# Supplementary material for: Assessing the Influence of Urine pH on the Efficacy of Ciprofloxacin and Fosfomycin in Immunocompetent and Immunocompromised Murine Models of Escherichia coli and Klebsiella pneumoniae Infection in the Lower Urinary Tract
Source: Antibiotics (Basel). 2024 Sep 1;13(9):827. doi: 10.3390/antibiotics13090827 (PMC11429092; doi:10.3390/antibiotics13090827)
Supplement: Supplementary file 1 [file antibiotics-13-00827-s001.zip › antibiotics-3164430-supplementary.pdf]

Assessing the Influence of Urine pH on the Efficacy of Ciprofloxacin and Fosfomycin in Immunocompetent and Immunocompromised Murine Models of *Escherichia coli* and *Klebsiella pneumoniae* Infection in the Lower Urinary Tract

SUPPLEMENTARY DATA

Figures S1 to S8

Tables S1 to S4

Supplementary Figure S1. Growth curves of *Escherichia coli* strains in MHB and urine, at pH 5 (pink), pH 7 (black), and pH 8 (purple).

(A) **Nu14**

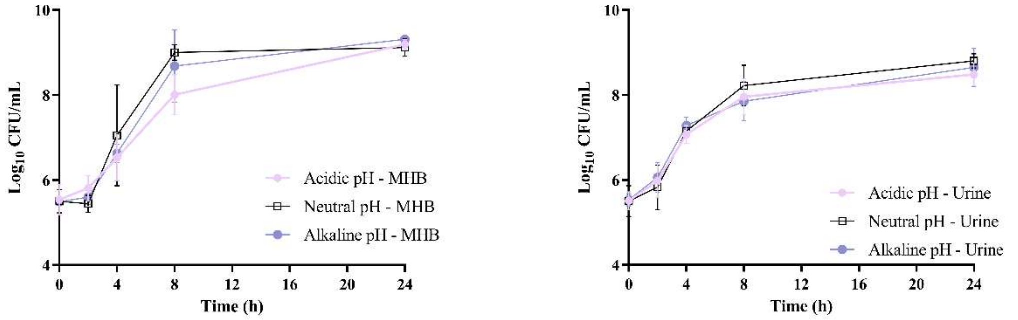

(B) **HUVR94**

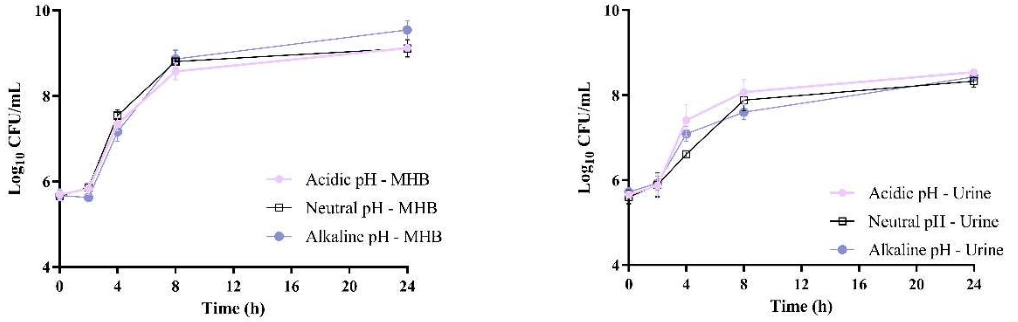

(C) **Nu14 *gyrA* D87G**

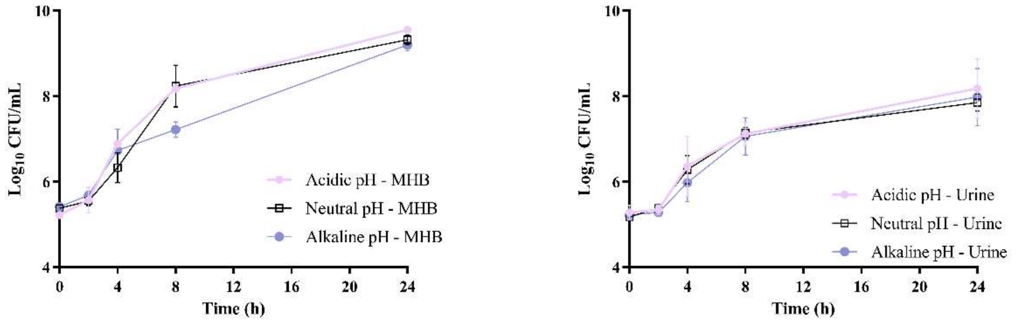

(D) **Nu14 *glpT***

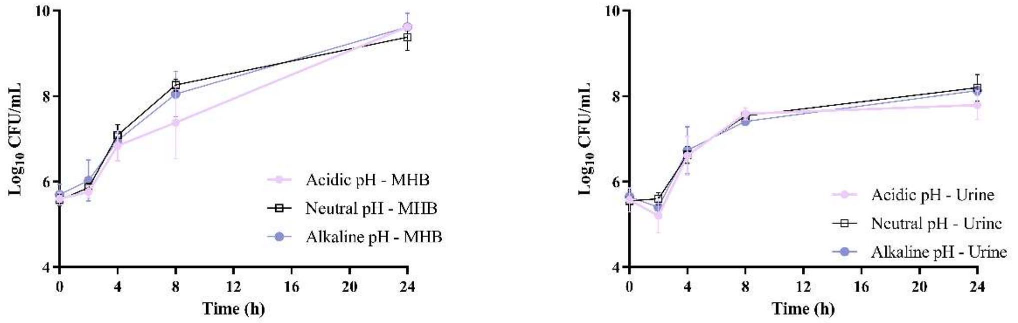

Supplementary Figure S2. Growth curves of *Klebsiella pneumoniae* strains in MHB and urine, at pH 5 (pink), pH 7 (black), and pH 8 (purple).

(A) **HUVR42**

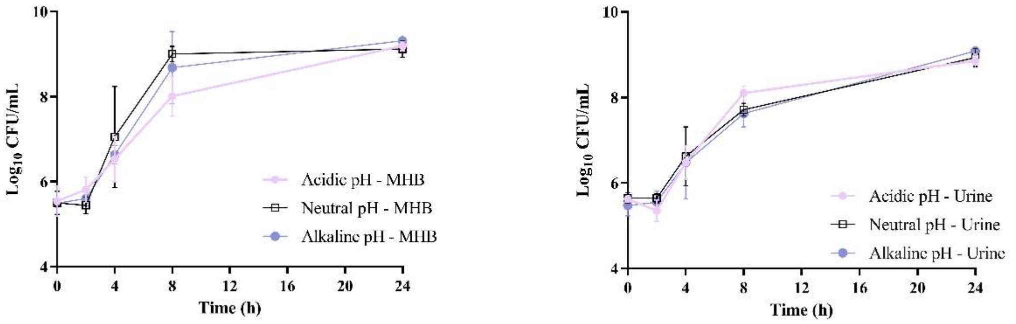

(B) **HUVR5**

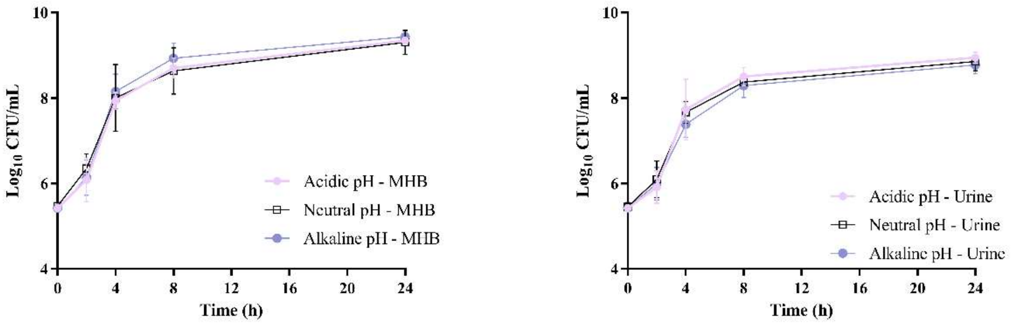

(C) **HUVR110**

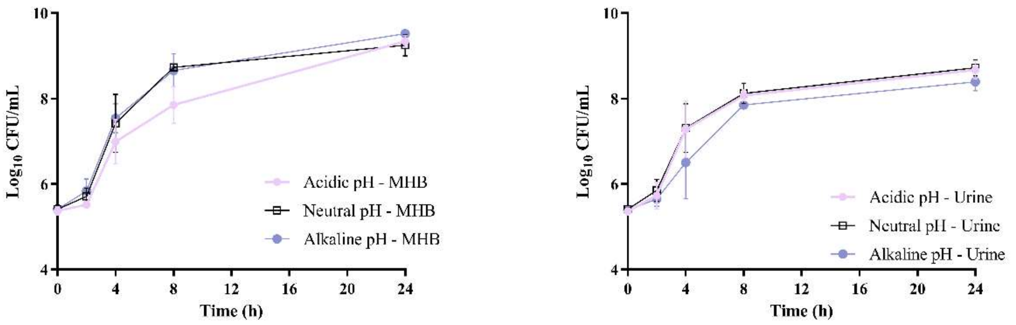

(D) **HUVR91**

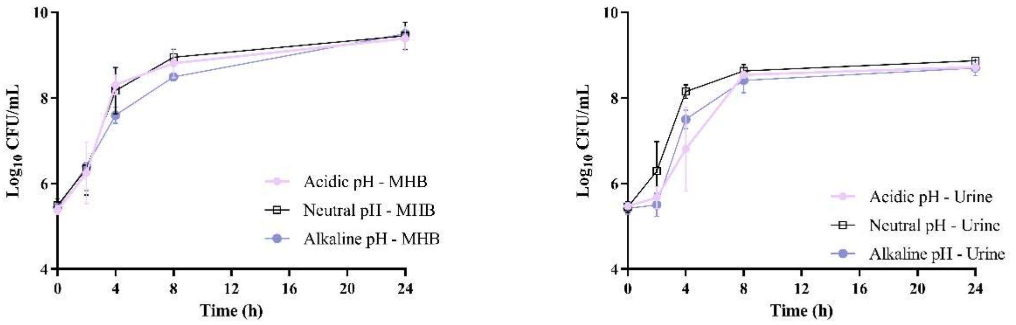

Supplementary Figure S3. *In vivo* efficacy of ciprofloxacin and fosfomycin for the experimental urinary tract infection in immunocompetent mice model by four *E. coli* strains at acidic (pink), neutral (white), and alkaline (purple) urine pH.

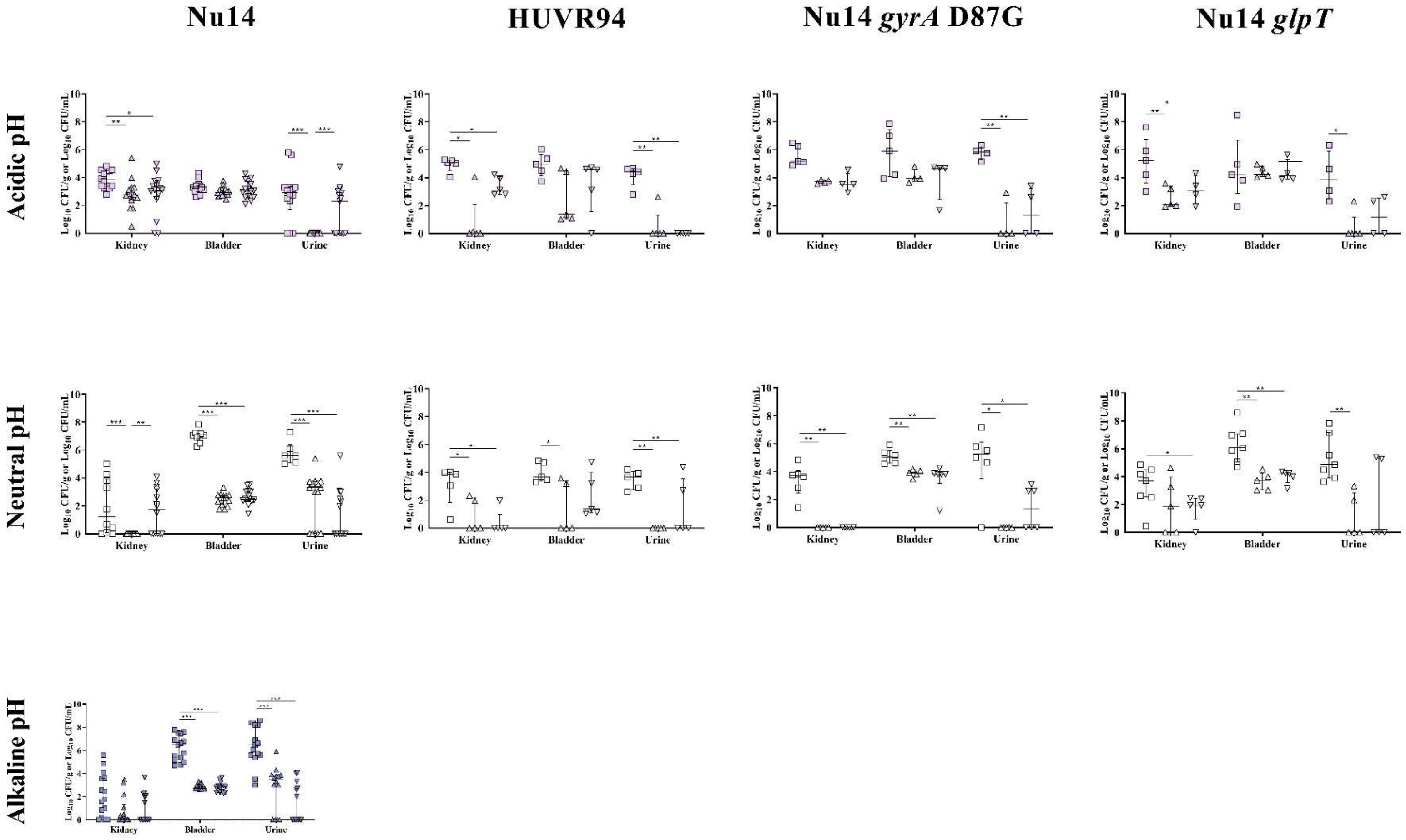

Square: Control group; Triangle: Ciprofloxacin-treated group; Inverted triangle: Fosfomycin-treated group. *: $P<0.05$; **: $P<0.01$; ***: $P<0.001$.

Supplementary Figure S4. *In vivo* efficacy of ciprofloxacin and fosfomycin for the experimental urinary tract infection in immunocompromised mice model by four *E. coli* strains at acidic (pink), neutral (white), and alkaline (purple) urine pH.

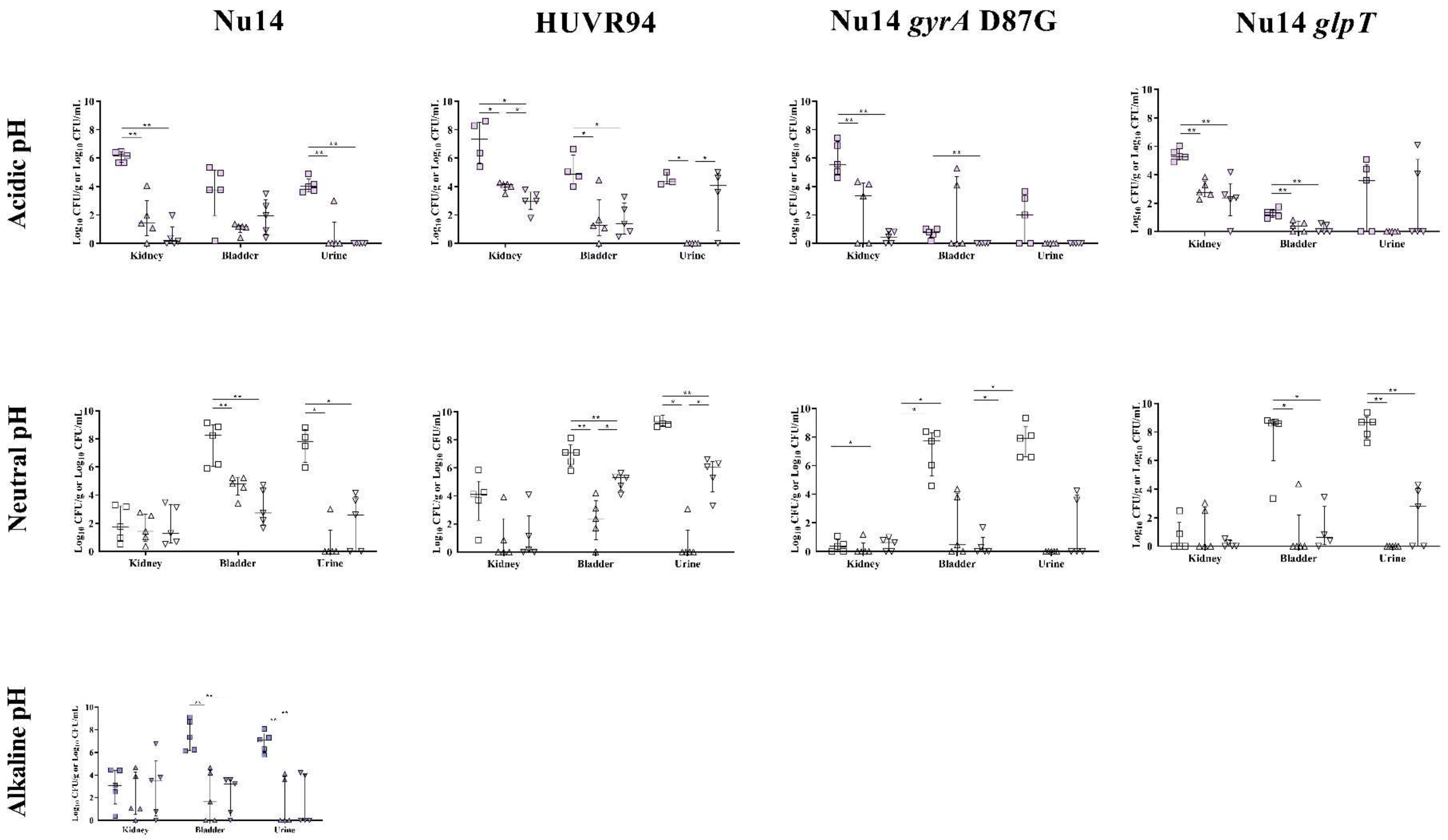

Square: Control group; Triangle: Ciprofloxacin-treated group; Inverted triangle: Fosfomycin-treated group. *: $P<0.05$; **: $P<0.01$; ***: $P<0.001$.

Supplementary Figure S5. Bloodstream infection and mortality rates in lower urinary tract infection models by *Escherichia coli* and *Klebsiella pneumoniae* strains, in immunocompetent and immunocompromised mice with acidic urine pH, at 72 hours of infection and treated with ciprofloxacin and fosfomycin.

(A)

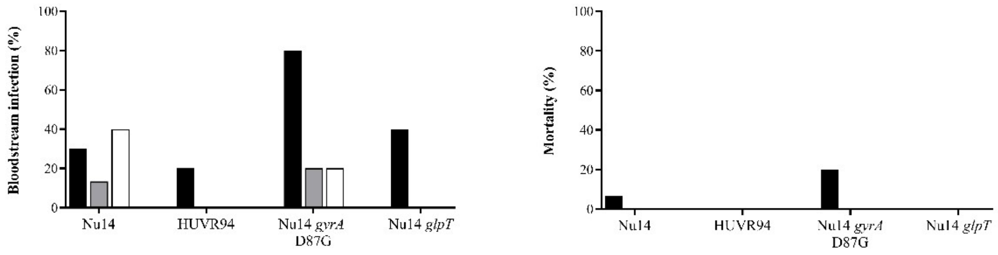

(B)

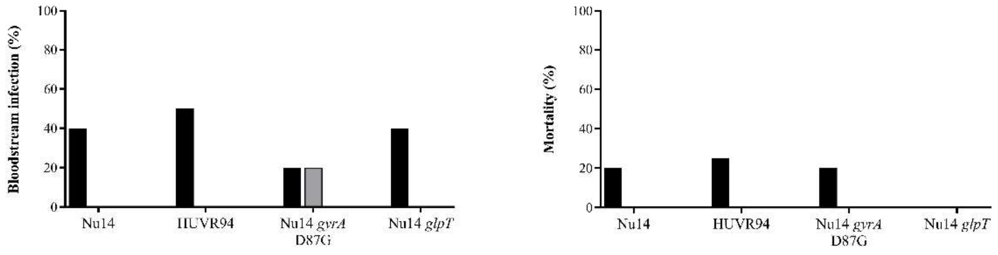

(C)

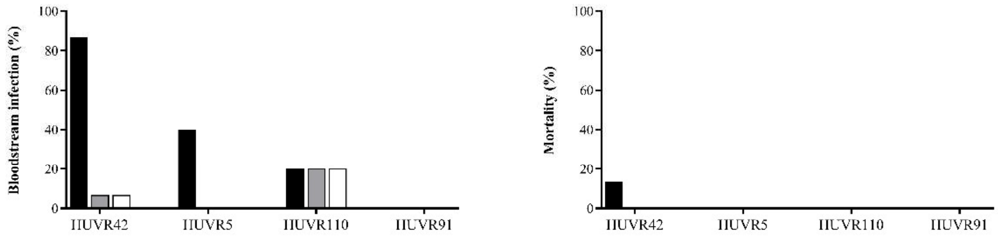

(D)

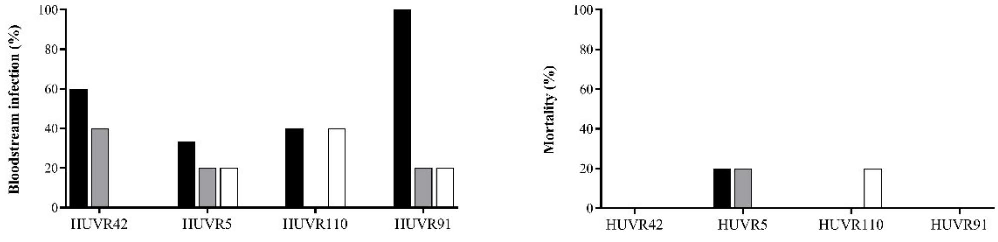

Bloodstream infection (left column) and mortality (right column) rates in lower urinary tract infection mice models by four *E. coli* strains: (A) Immunocompetent mice, (B) Immunocompromised mice, and by four *K. pneumoniae* strains: (C) Immunocompetent mice, (D) Immunocompromised mice. Black bars: Untreated mice groups; Grey bars: Ciprofloxacin-treated mice groups; White bars: Fosfomycin-treated mice groups.

Supplementary Figure S6. Bloodstream infection and mortality rates in lower urinary tract infection models by *Escherichia coli* and *Klebsiella pneumoniae* strains, in immunocompetent and immunocompromised mice with neutral urine pH, at 72 hours of infection, and treated with ciprofloxacin and fosfomycin.

(A)

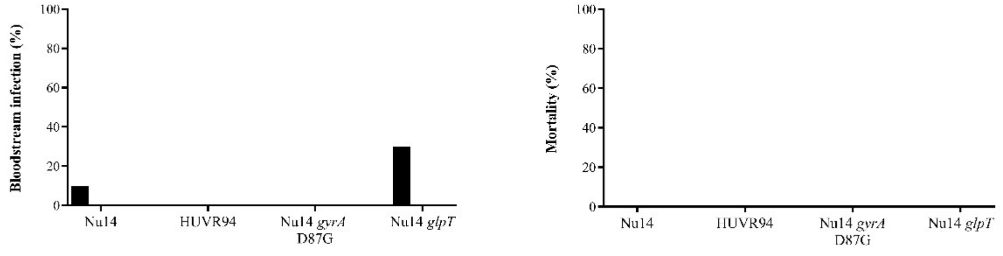

(B)

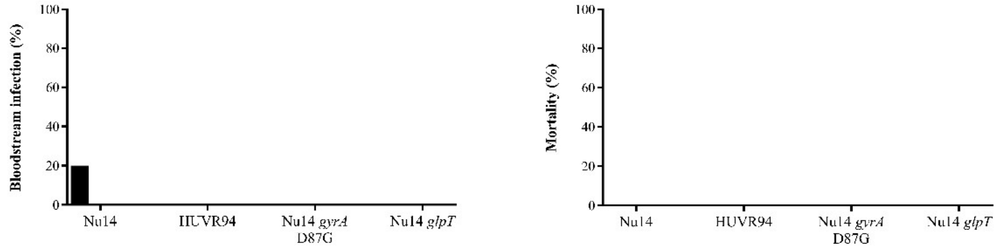

(C)

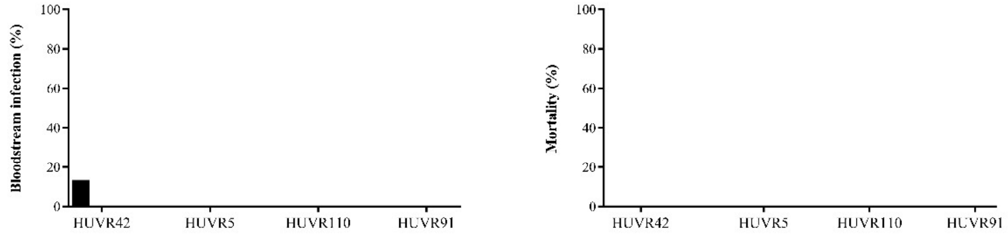

(D)

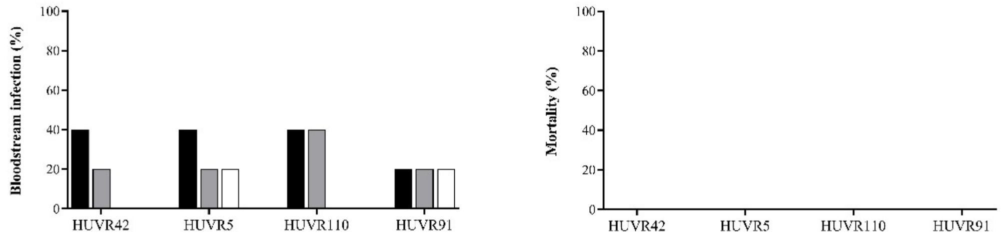

Bloodstream infection (left column) and mortality (right column) rates in lower urinary tract infection mice models by four *E. coli* strains: (A) Immunocompetent mice, (B) Immunocompromised mice, and by four *K. pneumoniae* strains: (C) Immunocompetent mice, (D) Immunocompromised mice. Black bars: Untreated mice groups; Grey bars: Ciprofloxacin-treated mice groups; White bars: Fosfomycin-treated mice groups.

Supplementary Figure S7. *In vivo* efficacy of ciprofloxacin and fosfomycin for the experimental urinary tract infection in immunocompetent mice model by four *Klebsiella pneumoniae* strains at acidic (pink), neutral (white), and alkaline (purple) urine pH.

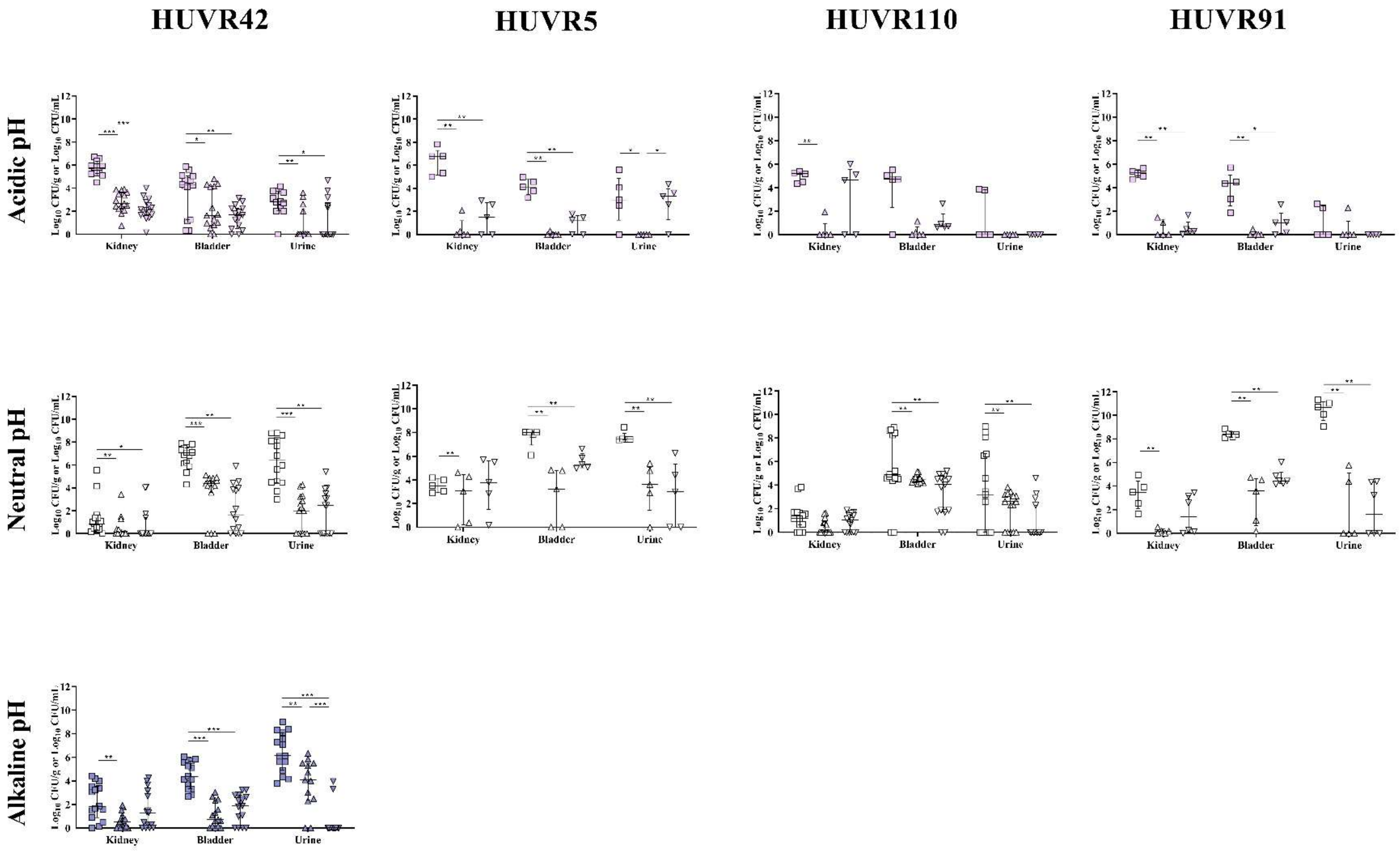

Square: Control group; Triangle: Ciprofloxacin-treated group; Inverted triangle: Fosfomycin-treated group. *: $P<0.05$; **: $P<0.01$; ***: $P<0.001$.

Supplementary Figure S8. *In vivo* efficacy of ciprofloxacin and fosfomycin for the experimental urinary tract infection in immunocompromised mice model by four *Klebsiella pneumoniae* strains at acidic (pink), neutral (white), and alkaline (purple) urine pH.

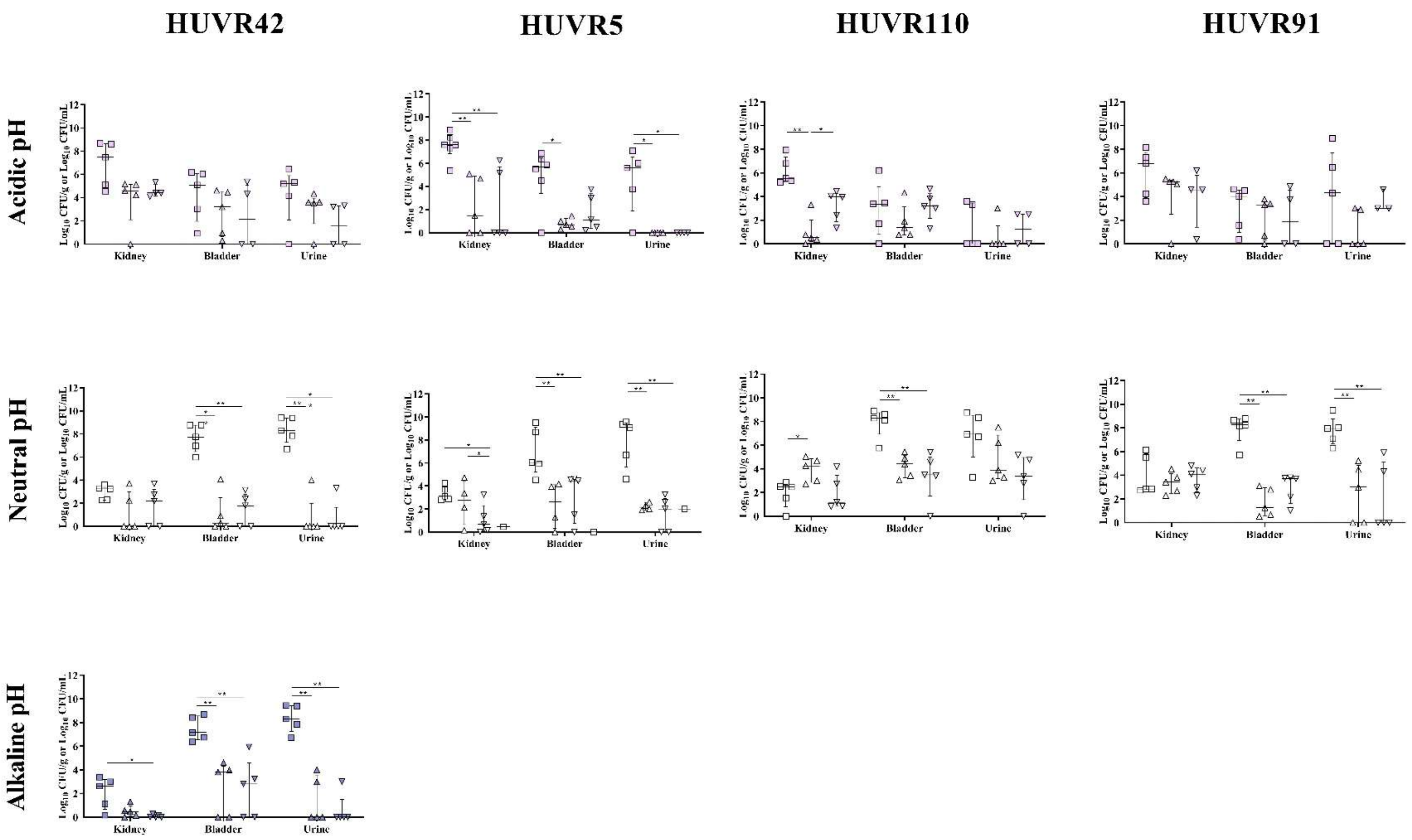

Square: Control group; Triangle: Ciprofloxacin-treated group; Inverted triangle: Fosfomycin-treated group. *: *P*<0.05; **: *P*<0.01; ***: *P*<0.001.

Supplementary Table S1. Pharmacokinetics of ciprofloxacin[#] and fosfomycin[##] in healthy C57BL/6J female mice following administration of a single intraperitoneal dose and pharmacodynamics for *Escherichia coli* Nu14, HUVR94, *gyrA*, and *glpT* and *Klebsiella pneumoniae* HUVR42, HUVR5, HUVR110, and HUVR91 strains.

**Drug**	**AUC_{0-24}** (mg·min /L)	**C_{max}** (mg/L)	**$T_{1/2}$** (h)	***E. coli* strains**			***K. pneumoniae* strains**		
				AUC_{0-24}/MIC	C_{max}/MIC	Δt/MIC	AUC_{0-24}/MIC	C_{max}/MIC	Δt/MIC
CIP (20 mg/kg ip route)	8.21	13.22	0.19	273.7 (Nu14)	440.7 (Nu14)	6.3 (Nu14)	1172.9 (HUVR42)	1888.6 (HUVR42)	27.1 (HUVR42)
				273.7 (HUVR94)	440.7 (HUVR94)	6.3 (HUVR94)	1.0 (HUVR5)	1.65 (HUVR5)	0.02 (HUVR5)
				32.8 (*gyrA*)	52.9 (*gyrA*)	0.76 (*gyrA*)	1.0 (HUVR110)	1.65 (HUVR110)	0.02 (HUVR110)
				821.0 (*glpT*)	1322.0 (*glpT*)	19 (*glpT*)	136.9 (HUVR91)	220.3 (HUVR91)	3.2 (HUVR91)
FOS (500 mg/kg ip route)	2695.35	1354.09	1.07	1347.7 (Nu14)	677.0 (Nu14)	0.5 (Nu14)	673.8 (HUVR42)	338.5 (HUVR42)	0.27 (HUVR42)
				5390.7 (HUVR94)	2708.2 (HUVR94)	2.1 (HUVR94)	21.1 (HUVR5)	10.6 (HUVR5)	0.008 (HUVR5)
				5390.7 (*gyrA*)	2708.2 (*gyrA*)	2.1 (*gyrA*)	673.8 (HUVR110)	338.5 (HUVR110)	0.27 (HUVR110)
				84.2 (*glpT*)	42.3 (*glpT*)	0.03 (*glpT*)	42.1 (HUVR91)	21.2 (HUVR91)	0.02 (HUVR91)

Ciprofloxacin (CIP); Fosfomycin (FOS); ip: intraperitoneal. AUC_{0-24}, area under the concentration–time curve; $fAUC_{0-24}$, free area under the concentration–time curve; C_{max}, maximum serum concentration in mice; $t_{1/2}$, terminal half-life; AUC $_{0-24}$ /MIC, ratio of AUC_{0-24} to minimum inhibitory concentration (MIC); C_{max}/MIC: ratio of maximum serum drug concentration to MIC; Δt/MIC: duration of time that drug concentration remains above the MIC during a dosing interval. [#] J Antimicrob Chemother 2013, doi: 10.1093/jac/dkt063. [##] Front Med 2021, doi: 10.3389/fmed.2021.615540.

Supplementary Table S2. Bacterial concentration in tissues and urine, and BSI and mortality rates at acidic urine pH conditions, in immunocompromised and immunocompetent mice after 72 hours of lower urinary tract inoculation by *Escherichia coli* and *Klebsiella pneumoniae* strains.

**Bacterial strain**		**Immunocompetent mice**		**Immunocompromised mice**		**P value[1]**
		N	Mean ± SD or percentage	N	Mean ± SD or percentage	
E. coli Nu14	Kidneys (Log_{10} CFU/g)	14	3.87±0.64	5	6.07±0.37	**<0.001**
	Bladder (Log_{10} CFU/g)	14	3.28±0.46	5	3.61±2.04	0.130
	Urine (Log_{10} CFU/mL)	14	2.70±1.79	5	4.07±0.52	**0.019**
	Bacteraemia (%)	14	29	5	40	1.000
	Mortality (%)	14	7	5	20	0.468
E. coli HUVR94	Kidneys (Log_{10} CFU/g)	5	4.91±0.51	4	7.16±1.54	**0.016**
	Bladder (Log_{10} CFU/g)	5	4.90±0.86	4	5.10±1.11	0.905
	Urine (Log_{10} CFU/mL)	5	4.15±0.78	3	4.51±0.44	1.000
	Bacteraemia (%)	5	20	4	50	0.524
	Mortality (%)	5	0	4	25	0.444
E. coli Nu14 *gyrA* D87G	Kidneys (Log_{10} CFU/g)	5	5.58±1.74	5	5.91±1.19	1.000
	Bladder (Log_{10} CFU/g)	5	5.77±1.61	5	0.72±0.34	**0.003**
	Urine (Log_{10} CFU/mL)	4	5.78±0.48	5	1.76±1.72	**0.016**
	Bacteraemia (%)	5	80	5	20	0.242
	Mortality (%)	5	20	5	20	1.000
E. coli Nu14 *glpT* missense mutation	Kidneys (Log_{10} CFU/g)	5	5.00±1.24	5	5.41±0.43	0.171
	Bladder (Log_{10} CFU/g)	5	4.68±1.83	5	1.29±0.31	**0.002**
	Urine (Log_{10} CFU/mL)	4	4.08±1.78	5	2.62±2.44	0.556
	Bacteraemia (%)	5	40	5	40	1.000
	Mortality (%)	5	0	5	0	1.000
K. pneumoniae HUVR42	Kidneys (Log_{10} CFU/g)	15	5.73±0.63	5	6.89±1.95	0.445
	Bladder (Log_{10} CFU/g)	15	3.71±1.92	5	4.24±2.24	0.395
	Urine (Log_{10} CFU/mL)	15	2.75±1.12	5	4.23±2.50	0.064
	Bacteraemia (%)	15	87	5	60	0.249
	Mortality (%)	15	13	5	0	1.000
K. pneumoniae HUVR5	Kidneys (Log_{10} CFU/g)	5	6.33±1.16	6	7.50±1.19	0.126
	Bladder (Log_{10} CFU/g)	5	4.13±0.69	6	4.85±2.50	0.177
	Urine (Log_{10} CFU/mL)	5	3.03±2.07	6	4.49±2.78	0.310
	Bacteraemia (%)	5	40	6	33	1.000
	Mortality (%)	5	0	6	16	1.000
K. pneumoniae HUVR110	Kidneys (Log_{10} CFU/g)	5	4.92±0.48	5	6.16±1.17	0.056
	Bladder (Log_{10} CFU/g)	5	3.98±2.25	5	2.95±2.31	0.421
	Urine (Log_{10} CFU/mL)	5	1.53±2.09	5	1.38±1.89	0.690
	Bacteraemia (%)	5	20	5	40	1.000
	Mortality (%)	5	0	5	0	-
K. pneumoniae HUVR91	Kidneys (Log_{10} CFU/g)	5	5.19±0.36	5	6.02±1.98	0.690
	Bladder (Log_{10} CFU/g)	5	3.88±1.46	5	3.02±1.93	0.690
	Urine (Log_{10} CFU/mL)	5	0.98±1.35	5	3.94±3.94	0.310
	Bacteraemia (%)	5	0	5	100	**0.008**
	Mortality (%)	5	0	5	0	-

P value[1]: Mann-Whitney U test was performed and Chi-square for qualitative ones.

Supplementary Table S3. Bacterial concentration in tissues and urine, and BSI and mortality rates at neutral urine pH conditions, in immunocompromised and immunocompetent mice after 72 hours of lower urinary tract inoculation by *Escherichia coli* and *Klebsiella pneumoniae* strains.

Bacterial strain		Immunocompetent mice		Immunocompromised mice		P value[1]
		N	Mean ± SD or percentage	N	Mean ± SD or percentage	
E. coli Nu14	Kidneys (Log_{10} CFU/g)	15	1.94±1.97	5	1.19±1.27	0.953
	Bladder (Log_{10} CFU/g)	15	7.02±0.43	5	7.67±1.53	0.594
	Urine (Log_{10} CFU/mL)	15	5.78±0.85	5	7.59±1.22	**0.038**
	Bacteraemia (%)	15	7	5	20	1.000
	Mortality (%)	15	0	5	0	**-**
E. coli HUVR94	Kidneys (Log_{10} CFU/g)	5	3.11±1.46	5	3.75±1.83	0.310
	Bladder (Log_{10} CFU/g)	5	4.00±0.72	5	6.92±0.88	**0.008**
	Urine (Log_{10} CFU/mL)	5	3.46±0.68	5	9.36±0.44	**0.008**
	Bacteraemia (%)	5	0	5	0	**-**
	Mortality (%)	5	0	5	0	**-**
E. coli Nu14 *gyrA* D87G	Kidneys (Log_{10} CFU/g)	5	3.39±1.16	5	0.40±0.45	**0.004**
	Bladder (Log_{10} CFU/g)	5	5.08±0.51	5	6.99±1.64	**0.082**
	Urine (Log_{10} CFU/mL)	5	4.69±2.46	5	7.71±1.14	**0.017**
	Bacteraemia (%)	5	0	5	0	**-**
	Mortality (%)	5	0	5	0	**-**
E. coli Nu14 *glpT* missense mutation	Kidneys (Log_{10} CFU/g)	5	3.25±1.52	5	0.66±1.07	**0.010**
	Bladder (Log_{10} CFU/g)	5	6.33±1.34	5	7.62±2.38	0.106
	Urine (Log_{10} CFU/mL)	5	5.33±1.60	5	8.37±0.84	**0.005**
	Bacteraemia (%)	5	20	5	0	0.470
	Mortality (%)	5	0	5	0	**-**
K. pneumoniae HUVR42	Kidneys (Log_{10} CFU/g)	15	1.30±1.54	5	2.94±0.62	**0.015**
	Bladder (Log_{10} CFU/g)	15	6.75±1.00	5	7.64±1.20	0.197
	Urine (Log_{10} CFU/mL)	15	6.34±2.04	5	8.33±1.14	0.070
	Bacteraemia (%)	15	14	5	40	0.249
	Mortality (%)	15	6	5	0	1.000
K. pneumoniae HUVR5	Kidneys (Log_{10} CFU/g)	5	3.51±0.56	5	3.33±0.62	0.690
	Bladder (Log_{10} CFU/g)	5	7.58±0.84	5	6.94±2.08	0.690
	Urine (Log_{10} CFU/mL)	5	7.62±0.46	5	7.86±2.16	0.690
	Bacteraemia (%)	5	0	5	40	0.444
	Mortality (%)	5	0	5	0	**-**
K. pneumoniae HUVR110	Kidneys (Log_{10} CFU/g)	5	2.30±1.34	5	1.88±1.15	0.841
	Bladder (Log_{10} CFU/g)	5	8.60±0.26	5	7.92±1.25	0.310
	Urine (Log_{10} CFU/mL)	5	7.75±1.11	5	6.80±2.15	0.841
	Bacteraemia (%)	5	0	5	40	0.444
	Mortality (%)	5	0	5	0	**-**
K. pneumoniae HUVR91	Kidneys (Log_{10} CFU/g)	5	3.29±1.25	5	4.01±1.68	0.548
	Bladder (Log_{10} CFU/g)	5	8.38±0.28	5	7.92±1.26	1.000
	Urine (Log_{10} CFU/mL)	5	10.43±0.89	5	7.76±1.19	**0.016**
	Bacteraemia (%)	5	0	5	20	1.000
	Mortality (%)	5	0	5	0	**-**

P value[1]: Mann-Whitney U test was performed and Chi-square for qualitative ones.

Supplementary Table S4. Bacterial concentration in tissues and urine, and BSI and mortality rates at alkaline urine pH condition, in immunocompromised and immunocompetent mice after 72 hours of lower urinary tract inoculation by *Escherichia coli* Nu14 and *Klebsiella pneumoniae* HUVR42 strains.

Bacterial strain		Immunocompetent mice		Immunocompromised mice		P value[1]
		N	Mean ± SD or percentage	N	Mean ± SD or percentage	
E. coli Nu14	Kidneys (Log_{10} CFU/g)	15	2.13±2.24	5	2.97±1.67	0.438
	Bladder (Log_{10} CFU/g)	15	6.22±1.08	5	7.51±1.37	0.190
	Urine (Log_{10} CFU/mL)	15	6.48±1.70	5	6.91±0.90	0.699
	Bacteraemia (%)	15	0	5	0	**-**
	Mortality (%)	15	0	5	0	**-**
K. pneumoniae HUVR42	Kidneys (Log_{10} CFU/g)	15	2.25±1.51	5	2.05±1.36	0.735
	Bladder (Log_{10} CFU/g)	15	4.51±1.18	5	7.48±1.03	**<0.001**
	Urine (Log_{10} CFU/mL)	15	6.42±1.69	5	8.58±0.75	**0.011**
	Bacteraemia (%)	15	13	5	60	0.073
	Mortality (%)	15	0	5	0	**-**

P value[1]: Mann-Whitney U test was performed and Chi-square for qualitative ones.
